# What Factors Influence the Interest in Working in the Public Health Service in Germany? Part I of the OeGD-Studisurvey

**DOI:** 10.3390/ijerph191811838

**Published:** 2022-09-19

**Authors:** Laura Arnold, Lisa Kellermann, Florian Fischer, Sophie Gepp, Franziska Hommes, Laura Jung, Amir Mohsenpour, Dagmar Starke, Jan M. Stratil

**Affiliations:** 1 Academy of Public Health Services, 40472 Duesseldorf, Germany; 2 Department of International Health, Care and Public Health Research Institute—CAPHRI, Faculty of Health, Medicine and Life Sciences, Maastricht University, 6211 Maastricht, The Netherlands; 3 German Network of Young Professionals in Public Health, 80539 Munich, Germany; 4 Institute of Public Health, Charité—Universitätsmedizin Berlin, 10117 Berlin, Germany; 5 Bavarian Research Center for Digital Health and Social Care, Kempten University of Applied Sciences, 87437 Kempten, Germany; 6 Charité—Universitätsmedizin Berlin, Corporate Member of Freie Universität Berlin and Humboldt-Universität zu Berlin, 10117 Berlin, Germany; 7 Division of Infectious Diseases and Tropical Medicine, Medical Faculty, Leipzig University, 04103 Leipzig, Germany; 8 Department of Population Medicine and Health Services Research, Bielefeld University, 33615 Bielefeld, Germany; 9 Department for Psychiatry, Psychotherapy and Psychosomatic Medicine, Vitos Kurhessen, 34308 Kassel, Germany

**Keywords:** public health workforce, public health services, health services administration, Germany, capacity building, job satisfaction, workforce research, workforce development, survey research methods, OeGD-Studisurvey

## Abstract

As in many European countries, the Public Health Service (PHS) in Germany has had considerable difficulties in attracting well-qualified personnel for decades. Despite ongoing political and societal debate, limited empirical research on possible causes and explanations is available. To identify areas of action, we explored reasons for the (lack of) interest in working in the PHS by conducting two cross-sectional surveys among 3019 medical students (MS), public health students, and students from other PHS-relevant fields (PH&ONM) in Germany right before (wave 1, 2019/2020) and during the COVID-19 pandemic (wave 2, 2021). While interest in working in the PHS among MS was low, it was considerably higher among PH&ONM. The prevalent underestimation of the importance of public health and low levels of knowledge about the PHS were identified as potential barriers. Although core activities of the PHS were often considered attractive, they were repeatedly not attributed to the PHS. A negative perception of the PHS (e.g., it being too bureaucratic) was prevalent among students with and without PHS interest, indicating that both a negative image and potentially structural deficits need to be overcome to increase attractiveness. Based on the findings, we propose approaches on how to sustainably attract and retain qualified personnel.

## 1. Introduction

The COVID-19 pandemic has revealed significant deficiencies in the public health system in Germany, particularly in the public health service (PHS, in German: Oeffentlicher Gesundheitsdienst) [1]. In addition to significant deficiencies in technical equipment and establishment of digital processes, the considerable lack of staff became apparent. The sharp increase in workload due to the pandemic had a serious impact on human resources and brought the PHS in Germany to its limits at almost all administrative levels. Although the shortage of well-qualified professionals had long been known and was widely problematized [2,3,4], it was not until the COVID-19-related (media) attention that the topic seemed to receive the necessary public consideration and was discussed by policymakers and decision makers [5,6,7,8,9,10].

### 1.1. Current Challenges for the Public Health Service Workforce in Germany

As a well-connected and largely prevention-oriented part of public administration, the PHS is—at least in theory—well suited to ensure the core public health activities [11], ranging from (non-)communicable disease control to the coordination of intersectoral efforts to prevent diseases and promote health through population-based approaches. Due to Germany’s federal structure, ensuring public health falls primarily within the responsibility of the 16 federal states, with different legislation [12]. While the states mainly provide the legal framework, much of the operational implementation is delegated to the municipalities, resulting in additional heterogeneity of tasks and accountabilities [13]. Therefore, the responsibility for delivering the core public health activities rests with approximately 375 local health authorities (LHA). Because of their local embedding, LHAs can play an important role in improving population health and addressing social inequalities by ensuring the delivery of the 10 Essential Public Health Operations (EPHOs) on local level, as recommended by the World Health Organization (WHO) in the European Action Plan for Strengthening Public Health Capacity and Services [14].

The huge impact of population-based public health interventions is reflected in the nearly steady increase in life expectancy over the past 120 years, largely because of the control of infectious diseases, more abundant and safer foods, improved hygiene and sanitary conditions, as well as broad economic growth and rising living standards [15,16,17,18]. Therefore, it is all the more surprising that despite this success, the PHS is often not seen as an attractive field to work in [19,20]. This seems to be especially true for LHA, the largest public health actor in the public health system in Germany in terms of number of employees [21,22], which have hardly succeeded in recruiting young professionals in recent years. Small rural LHA in particular have difficulties finding well-trained professionals and are, therefore, confronted with a significant shortage of personnel [23,24,25,26]. In view of the high average age of employees in the public sector and the fact that many positions are already vacant, it can be assumed that the challenges in recruiting new staff will become even more pressing in the forthcoming years [27,28,29].

Various factors have been discussed as possible reasons for the often-perceived low attractiveness of the PHS, including, among others, insufficient career opportunities, lack of approaches to continuous professional development, fixed hierarchical structures, low salary, and one-sided workload [30,31,32,33]. Moreover, there is a low appreciation for administrative activities in the public sector in general—which is also reflected in the international literature [34]—and a negative image of the PHS in Germany as being too bureaucratic, rigid and inflexible among students and young professionals [35,36]. In addition, it can be assumed that until the outbreak of the COVID-19 pandemic, the PHS was largely unknown to the public in Germany and many students of PHS-relevant disciplines, such as medicine or public health. Students and young professionals were neither aware of the existence of the PHS as a potential employer nor did they have concrete ideas about its far-reaching impact and responsibilities.

In this context, the lack of integrated population-based public health approaches and topics in PHS-relevant study programs has been criticized several times [37,38,39]. A content analysis of central teaching and examination documents from 2019 revealed that numerous topics essential for the work of the PHS, such as solid knowledge about health inequalities and social determinants of health (SDH), were hardly embedded in the medical curricula in Germany [40]. Low visibility of the PHS within study programs could also contribute to students underestimating the relevance of public health approaches in general and thus also misclassifying the effectiveness and reach of the work of the PHS.

Another reason for the low attractiveness of working in the PHS could be the perceived stronger orientation of the PHS towards medical professions, which has been questioned and criticized by students and young professionals in Germany several times [31,41,42]. To date, many approaches to increase the attractiveness of the PHS have focused primarily on medical students and physicians [43,44], while graduates from other fields of studies have rarely been addressed.

### 1.2. The OeGD-Studisurvey

Since, to our knowledge, there are no empirical data on the perceived attractiveness of the PHS in Germany, we have set up the OeGD-Studisurvey, a comprehensive research project to explore and analyze the perception of the PHS in Germany as potential employer among medical students, public health students and students of other study programs with potential PHS-relevance. The research project was conducted by the German Network of Young Professionals in Public Health (NOEG), the Academy of Public Health Services (AOEGW), the Federal Association for Physicians in the Public Health Service (BVOEGD), and the German Medical Students’ Association (bvmd).

Within the scope of the OeGD-Studisurvey, we aim to investigate reasons for the perceived low attractiveness of the PHS in Germany as a potential employer among students from PHS-relevant fields of studies in order to derive possible solutions for an appealing PHS in the future and by this strengthen the PHS workforce. 

**Figure 1:**
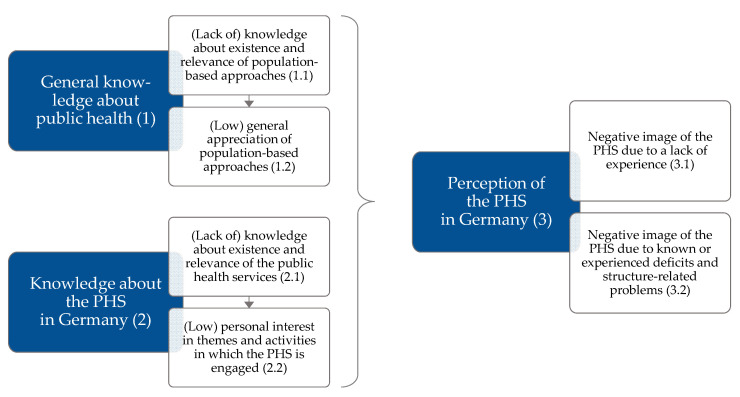
Logic model on potential causes and pathways of the perceived low attractiveness of the public health service (PHS) and an occupation within it.

We will do so by analyzing factors that can potentially explain the interest in working in the PHS in Germany among students, based on a theoretically derived logic model, visualized in Figure 1.

Various causes and pathways have to be considered when exploring the perception of the PHS in Germany among students. For instance, a lack of knowledge about the existence and relevance of population-based public health approaches focusing on upstream (often social) determinants of health to ensure health equity and improved working and living conditions [45,46,47] could play an important role in the perceived low attractiveness (Figure 1: 1.1). Similarly, perceived low effectiveness and relevance of public health approaches in general (Figure 1: 1.2) or an insufficient linkage of these approaches with tasks and activities of the PHS (Figure 1: 2.1), could contribute to this. It is also conceivable that students might just not be interested in core topics and functions of the PHS and the resulting tasks and activities that working in the PHS would involve (Figure 1: 2.2).

All of these factors could affect the perception of the PHS as a prospective employer (Figure 1: 3). Barriers that may hinder individuals from pursuing a career in the PHS in Germany can be divided into two groups: Perceptions of the PHS may be hampered due to unverified stereotypes that could be overcome, for example, through factual knowledge and experience (Figure 1: 3.1). Perceived low attractiveness may also result from known or experienced deficits and structure-related problems of the PHS that cannot be easily overcome, as they would require fundamental changes within the PHS (Figure 1: 3.2).

Building on the logic model, we will first analyze the general interest in working in the field of public health among students of PHS-related programs to identify contributing factors. Based on this, we will assess the general knowledge about public health measures and its potential influence on population health as well as the knowledge about the PHS in particular. Afterwards, we will determine how the PHS is perceived among students before we will explore potential targets to improve the attractiveness of the PHS as a prospective employer. Barriers to entry in the PHS workforce and solutions to strengthen public health recruitment in Germany from a young professional’s perspective are described in an accompanying paper [48].

The OeGD-Studisurvey thereby provides the first empirical data on the perception of the PHS in Germany as a potential employer among both medical and PH & ONM students. In assessing interest in the PHS, exploring the reasons underlying difference in interest (and the lack thereof) as well as by identifying potential barriers and facilitators, our objective is to provide a basis for evidence-informed decision making on how to sustainably improve recruitment and retention of qualified employees for the PHS in the future.

## 2. Materials and Methods

Two comprehensive cross-sectional surveys were conducted right before and during the COVID-19 pandemic, to assess the perceptions and expectations of students in PHS-relevant fields of study about their potential future employers in Germany. The first survey (wave 1) was conducted from early December 2019 to early April 2020 and focused on wishes and expectations of students from PHS-relevant fields of study programs regarding their future jobs and employers. The second survey (wave 2) was conducted from beginning of June to end of September 2021. This time frame was chosen in order to identify changes that may have resulted from the newfound attention on the PHS during the COVID-19 pandemic. Changes in the perception of the PHS as a potential employer from a student’s perspective was a focus of the second wave. Both surveys addressed the same target group and were based on the same sampling procedure. However, two independent samples were drawn due to the cross-sectional study design.

### 2.1. Study Population and Sampling Procedure

Students from PHS-relevant and accredited advanced degree study programs at state or state-recognized German universities were eligible to participate. Among others, this included all medical degree programs as well as all public health degree programs, health science degree programs, and degree programs in natural and administrative sciences potentially preparing students for the PHS.

Since there is no institution in Germany through which the students of all OeGD-relevant study programs can be reached directly, we contacted the secretariats of all relevant study programs and institutions with the request to forward an invitation letter to all students. In addition, relevant institutions and stakeholders in the field of public health, such as the NOEG, the AOEGW, the BVOEGD, the bvmd, and the Institute for Medical and Pharmaceutical Examination Questions (IMPP), were asked to draw attention to the survey via their distribution lists and to forward the invitation to the survey to interested persons. Finally, the invitation to participate was shared via social media channels of the NOEG and the bvmd. Three weeks later, all recipients received an email reminder to boost participation in the survey.

**Figure 2:**
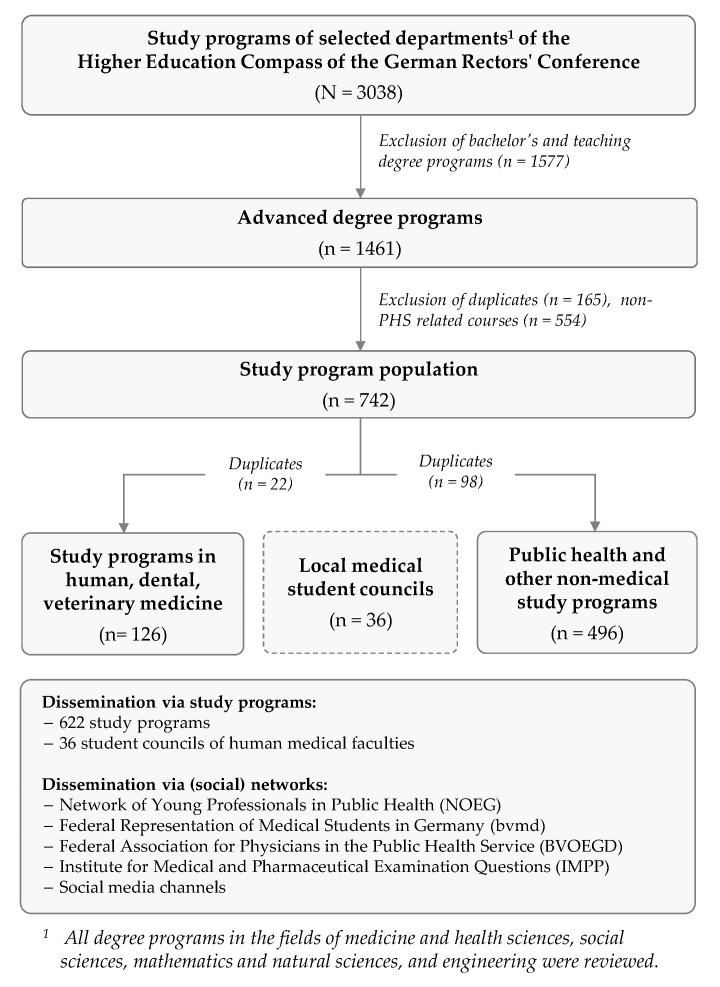
Flowchart for obtaining the distribution list to generate the OeGD-Studisurvey sample.

The corresponding e-mail addresses of the secretariats were identified specifically for this purpose, based on a systematic extraction of relevant study programs from the Higher Education Compass, a public database of the German Rectors’ Conference (HRK, in German: Hochschulrektorenkonferenz) that publishes information from state and state-recognized German universities about their study and doctoral opportunities [49]. The process to identify PHS-relevant study programs is shown in Figure 2.

In a two-stage procedure, all degree programs in the fields of medicine and health sciences, social sciences, mathematics and natural sciences, and engineering were selected in October 2019 (*N* = 3038). After excluding all bachelor’s and teaching degree programs (*n* = 1461), the remaining advanced degree programs were examined by two authors (LA, DS) with regard to their appropriateness to PHS-related fields of activity. Disagreements or uncertainties regarding eligibility were resolved by discussion among the two authors. After excluding duplicates, the final distribution list included 622 study programs: 126 with a medical focus and 496 with a non-medical focus. The complete distribution list can be seen in Supplementary Materials. 

The HRK is a voluntary association of state and state-recognized universities in Germany and currently has 269 member universities, at which more than 90% of all students in Germany are enrolled [50]. We therefore assume that we have covered a large part of all OeGD-relevant study programs in Germany through the chosen approach.

### 2.2. Survey Instrument and Data Collection

Two self-designed questionnaires were administered, developed by a multi-professional working group consisting of members of the NOEG, AOEGW, BVOEGD, and bvmd in an iterative, interdisciplinary process. The online-based questionnaires were generated using SoSci Survey version 3.2.00 and made available via www.soscisurvey.de [51].

A pretest with 15 participants was conducted to test the comprehensibility of questions and completion time. Necessary amendments were subsequently made. The survey was reviewed by the ethics committee of Bielefeld University and all participants were asked to provide informed consent in electronic format.

The two online-based questionnaires contained open-ended as well as multiple-choice questions. The questionnaire of wave 1 comprised six sections: Section I consisted of 10 items eliciting sociodemographic data from the respondents. Section II focused on recording personal expectations about working life and contained a five-level rating question with 20 items. Section III was designed to assess the level of knowledge about public health among the participants by means of six items. Sections IV and V recorded personal interest in the professional field of public health as well as activities in the PHS with three and one item, respectively. In Section VI, associations and polarities about the perception and image of the PHS among students were recorded. Contrasts in the perception of the PHS were surveyed as a polarity profile, providing fixed categories. Within the scope of several open-ended questions, we asked for arguments for and against the PHS as a potential employer, as well as corresponding suggestions for increasing its attractiveness. The results of these free-text statements are the subject of the accompanying paper [48].

We expected that the PHS had become better known in the course of the COVID-19 pandemic, where many students had been employed in testing and contact tracing activities for the PHS [52]. Thus, the questionnaire used in wave 1 was adapted for wave 2. Section III was removed, and previous experience was included in the context of activities in the PHS. Potential opportunities for the further development of the PHS to increase its attractiveness were also addressed. Both questionnaires can be seen in Supplementary Materials. 

After project completion, the quantitative data of the OeGD-Studisurvey will be available on https://osf.io/uxftz. To prevent identification of individuals through the combination of the open-ended answers and socio-demographic data, the unredacted dataset will not be publicly available due to privacy reasons. The data presented in this study are available on request from the corresponding author.

### 2.3. Analysis

In order to investigate to what extent a relevant proportion of students consider the tasks and activities of the PHS to be important and relevant, while rating a career in the PHS as not an option, a multi-layered analysis approach was applied according to the logic model (Figure 1). Based on the classification of the personal interest in working for the PHS in Germany, we assessed factors potentially explaining this interest stratified by the participants’ knowledge about public health approaches (1.1) and its assumed impact (1.2). Interest in (2.2) and knowledge about (2.1) specific topics, tasks, and activities of the PHS were analyzed by comparing reported PHS knowledge with its anchoring in the curriculum and general interest in a related occupational activity in that same field. Next, the perception of the PHS was assessed using a multilevel polarity profiling of characteristics associated with the PHS (3). Finally, potential targets to improve the attractiveness of the PHS as prospective employer were identified by examining factors that young professionals assessed as generally relevant for their future work life and career.

Univariate and bivariate analyses were conducted with the quantitative items capturing the attractiveness of the PHS as a prospective employer. For this purpose, unadjusted comparisons were made between medical students who indicated a high preference for future employability within the PHS and medical students with a low preference, using Pearson’s Chi^2^ test (or Fisher’s exact test, as appropriate) for categorical and Student’s *t*-test for continuous variables. The same comparisons were made for public health students and students form other non-medical fields of study with potential relevancy for the PHS. When appropriate, we also differentiated between wave 1 and wave 2 to capture any changes in perceptions during the COVID-19 pandemic. Due to the gender imbalance in medical-related courses and courses relevant to working in the PHS [53,54], comparisons were not made separately for men and women.

Collected data from SoSci Survey were exported into an excel spreadsheet (Microsoft Excel, 2013) for data cleaning and coding of the open-ended questions. All quantitative analyses were performed with R version 4.0.3 [55]. For all analyses, a *p*-value < 0.05 was considered to be statistically significant. Analysis and description followed the STROBE guidelines for observational studies [56].

## 3. Results

### 3.1. Characteristics of the Study Population

A total of 2450 students participated in wave 1 and 569 in wave 2. The majority of all 3019 respondents were females (74%), with a mean age of 25.0 years (±5.3 SD). Medical students accounted for 68% of the respondents, students of public health for 15% and students of other studies qualifying for the PHS, such as sociology, social work, biology, engineering or public administration, for 17%. Due to the unequal group sizes, we will distinguish between medical students (MS) and public health and other non-medical (PH&ONM) students in the following. Key characteristics of the included study participants are provided in Table 1.

As expected, considerably more women than men took part in both waves. The study population was slightly younger in wave 1 (24.6 years, ±4.8 SD) than in wave 2 (26.1 years, ±6.8 SD), with students who reported a general interest in working in the PHS tending to be older than students without such an interest. Fewer medical students participated in wave 2, in particular fewer medical students without interest in working in the PHS. Overall, 17% of all participants stated to have a second degree, i.e., being enrolled in another minor or having completed an additional degree in advance, with students with PHS interests again more prevalent. In wave 2, we also asked whether students had gained initial work experience in the PHS in Germany, such as during an internship or part-time employment. Students with experience were more likely to show general PHS interest than students without prior experience. Due to the cross-sectional study design, however, no further (especially causal) interpretations are permitted in this regard.

### 3.2. General Interest in Working for the PHS in Germany

Overall, 13% of the participants reported that they can generally imagine themselves working in the PHS. This general PHS interest was much more pronounced among PH&ONM students than among medical students in wave 1 and 2 (Table 2). Considerably fewer students could imagine working in the PHS at the municipal level than at the federal or national level, with apparent differences between medical students and PH&ONM students.

While 27% of all medical students and 66% of all PH&ONM students could imagine working in the PHS in general (“yes” or “rather yes”) in wave 1, this was an option for 37% of the medical students and even 81% of the PH&ONM students in wave 2. More medical students could imagine working at the federal or national level in wave 2 (50%) than in wave 1 (29%), while the proportion among PH&ONM students was about the same in both waves. In order to distinguish between students with and without general PHS interest in the following analysis, all statements of general interest that participants answered “yes” or “rather yes” were subsumed into the binary index “PHS interest”.

### 3.3. Interest in Working in a PHS-Related Field

**Figure 3:**
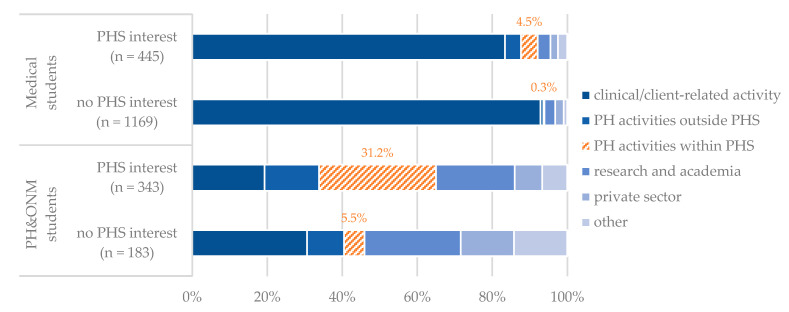
Preferred field of activity after graduation among medical (*n* = 2058) and PH&ONM students (*n* = 961) in Germany, according to their PHS interest in wave 1 of the OeGD-Studisurvey (The proportion of students who ranked a public health (PH) activity first is highlighted in orange).

In wave 1, we explored the preferred field of work after graduation by asking all participants to rate various areas of work according to their personal preference. The majority of participants ranked a clinical, client-related activity on top (74%), research and academia were prioritized by 8% and public health activities within the PHS by 7%. Of all medical students with PHS interest, 83% prioritized a clinical, client-related activity compared to 93% of medical students without PHS interest. Among PH&ONM students with PHS interest, 19% prioritize a clinical, client-centered activity compared to 31% with no interest. A public health-related activity within the PHS was prioritized by 31% of PH&ONM students with PHS interest and 5% of the medical students with PHS interest (Figure 3, more details can be found in Table A1 in Appendix A).

The results from wave 1 were validated in wave 2 by surveying the preferred fields of activity after graduation using a four-point Likert scale ranging from “No, definitely not” to “Yes, definitely”. While every second PH&ONM student with PHS interest could imagine a public health-related activity within the PHS in wave 2 (49%), this was an option for only one in four medical students with PHS interest (24%). The proportion of those who could envision a clinical, client-related work was correspondingly lower. More details can be found in Table A2 in Appendix A.

### 3.4. General Knowledge about Population-Based Public Health Approaches

To identify factors that might be related to an interest in working in the PHS in the future, we surveyed differences in knowledge about public health in general and collected data on the perceived impact of various public health interventions, according to part 1 of the logic model (Figure 1).

As a proxy for existing knowledge about public health in general, participants were asked in several multiple-choice questions to estimate trends in life expectancy (LE) in high- and low-income countries, to estimate differences in LE between groups of high and low socioeconomic status, and to estimate LE differences between men and women. While the average shift in LE in high-income countries over the last 30 years was answered correctly by almost 75% of all participants (with no apparent difference between medical and PH&ONM students, regardless of PHS interest), the situation in low- and middle-income countries seemed to be unknown for the broad majority, as only 10% answered this question correctly (again, with negligible differences between medical and PH&ONM students). While differences in LE among women with low and high socio-economic status (SES) were correctly estimated by one out of three participants (29%), only about one out of ten correctly assessed differences in LE between men with low and high SES. No notable differences between MS and PH&ONM students were found in this regard.

Medical students with PHS interest estimated the average difference in LE between women and men more often correctly (60%) than MS without interest (54%). Other than that, we found no differences between students with and without PHS interest. See Appendix A Table A3 for more details. Overall, about 37% of all study participants answered only one of five questions correctly, about one-fifth estimated at least three questions correctly (22%), and at least four correct answers were given by a mere three participants. We found no relevant difference in the number of correct responses between medical students and PH&ONM students with and without PHS interest.

In order to assess the perceived importance of public health measures, participants were asked to rank a broad variety of public health measures and interventions according to their estimated relevance for improving LE in Germany over the past 120 years. Medical therapy of infectious diseases in terms of antibiotics was ranked among the top three by more than two thirds of all medical students with PHS interest (70%) as well as all PH&ONM students (66%), followed by primary medical prevention (58% of MS and 48% of PH&ONM students with PHS interest), and measures to reduce poverty or malnutrition (55% of MS and 47% of all PH&ONM students with PHS interest). Non-medical preventive measures and health promotion measures, such as smoking bans or measures to increase health literacy, were prioritized by 16% of all participants with almost identical proportions between MS (17%) and PH&ONM students with PHS interest (18%). Minor importance was also attributed to secondary prevention measures, such as cancer screening, by 15% of all interested MS and 20% of all interested PH&ONM students.

More PH&ONM students without PHS interest rated primary medical prevention measures as relevant (64%) than PH&ONM students with PHS interest (48%). Besides that, we found no relevant differences regarding the assumed public health impact between medical students and PH&ONM students with and without PHS interest. See Appendix A Table A4 for more details.

### 3.5. Knowledge about and Interest in Specific Topics, Tasks and Activities of the PHS in Germany

To analyze the lack of knowledge about PHS-relevant tasks and activities as postulated in part 2 of the logic model (Figure 1), associations with and attributions to six core public health fields of activities were surveyed in wave 1. Based on three questions, all participants were asked if they associate the given fields of activity with the PHS, to what extent the fields of activity were part of their studies, and whether the activities are suitable for themselves in the future. The main results are briefly summarized below, more detailed information can be found in Table 3.

Primary and secondary prevention and supporting vulnerable groups were not associated with the PHS by 33% of participants each. Minor differences were notable between medical students without PHS interest, who were less likely to attribute supporting measures, prevention measures, and health protection measures to the portfolio of the PHS than MS with PHS interest.

The curricular anchoring of the six public health core areas varied greatly: While (medical) primary and secondary prevention was reported to be intensively covered within medical (61%) as well as PH&ONM study programs (52%), outbreak and crisis management was hardly present in both. Health planning and policy advice were stated as intensely covered by 31% of all PH&ONM students compared to 2% of all medical students. Working with vulnerable groups, and behavioral and primary prevention interventions were also more often reported as intensively covered by PH&ONM students. Health protection and infection disease prevention and (medical) primary and secondary prevention, on the other hand, were more frequently indicated as addressed intensively within the medical curriculum (Table 3).

Students with PHS interest were more likely to state that one of the six core public health fields would be an option for them compared to students with no interest. Primary and secondary prevention were by far the most commonly considered by medical students interested in the PHS (81%), followed by working with vulnerable groups (70%). Among PH&ONM students behavioral and primordial prevention, support of vulnerable groups and health planning and policy advice were considered most frequently.

### 3.6. Interest in Core Public Health Fields of Activities Regarding Their Curricular Coverage and Attribution to the PHS in Germany

Interest in the six core areas was also compared with the extent to which the participants reported that the topics were intensively covered during their main studies and attributed to the PHS in Germany.

Relevant differences emerged in particular in the areas of health planning and policy advice, outbreak and crisis management, and supporting vulnerable groups: In all three areas of activity, medical students, for whom the occupational fields were an attractive option after graduation, reported at the same time a low level of anchoring in their studies (Table 4). A large proportion of PH&ONM students with an interest in working in outbreak and crisis management reported marginal anchoring in their studies.

About one third of students interested in primary or secondary prevention or in supporting vulnerable groups do not attribute these activities with the PHS. In the areas of outbreak and crisis management, health planning/policy advice, and behavioral and primary prevention, the proportion was about 25% each, with no noticeable differences between medical students or PH&ONM students (Table 4).

### 3.7. Perception of the PHS

To assess part 3 of the logic model (Figure 1), characteristics associated with the PHS were collected using multilevel polarity profiling in waves 1 and 2. All of the in total eight contrasting juxtapositions could be assigned to five levels, so that all participants could choose which expression (e.g., ranging from interesting, diverse, and exciting to boring and monotonous) they most closely associated with the PHS.

**Figure 4:**
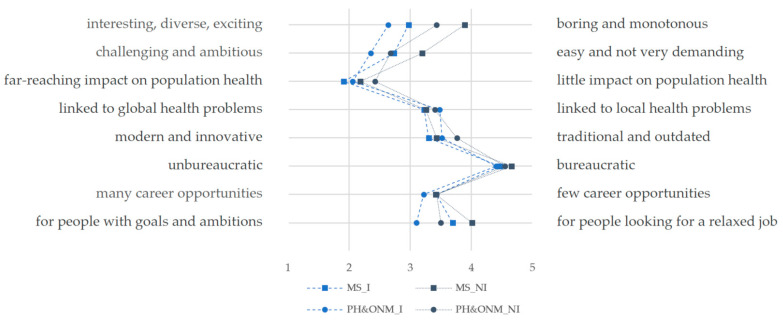
Polarity profile of characteristics associated with the PHS by field of study and general PHS interest of students in Germany, OeGD-Studisurvey (Abbreviations: MS_I = medical students with PHS interest, MS_NI = medical students without PHS interest, PH&ONM_I = PH&ONM students with PHS interest, PH&ONM_NI = PH&ONM students without PHS interest).

Both medical students and PH&ONM students with PHS interest were more likely to characterize the PHS as interesting, diverse and exciting, to consider PHS-related tasks and activities as challenging and demanding, and to attribute a high overall impact on population health to the tasks and activities of the PHS compared to students without PHS interest (Figure 4).

**Figure A1:**
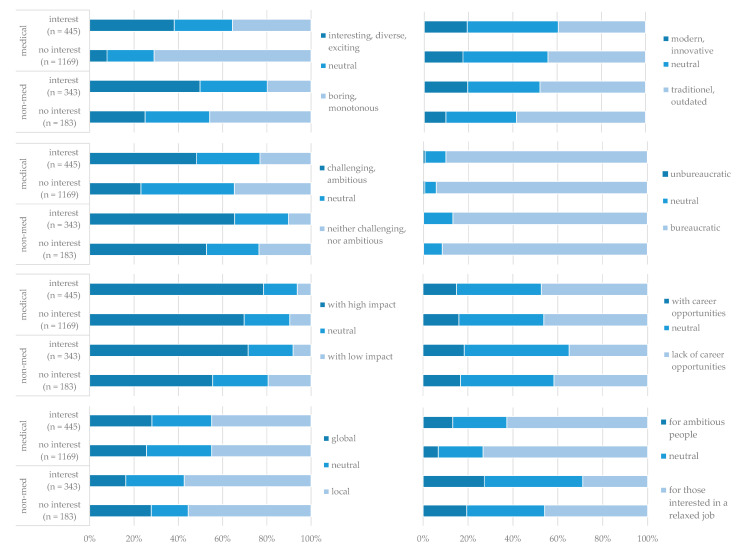
Relative proportion of attributes and characteristics associated with PHS. Shown are the results of a polarity profile based on eight contrasting comparisons differentiated by field of study and general PHS interest wave 1 and wave 2, OeGD-Studisurvey.

Almost regardless of the general PHS interest, the vast majority of all students characterized the PHS as highly bureaucratic (93% of all medical students and 89% of all PH&ONM students). Furthermore, the PHS was more likely to be associated with individuals who were interested in a relaxed job than with people with goals and ambitions. The latter is much more pronounced among medical students with PHS interest (63%) than among interested PH&ONM students (33%). PH&ONM students were slightly more positive in their characterization of the PHS than medical students, as well as students with general PHS interest. More detailed information can be found in Figure A1 and Table A5 in Appendix A.

To account for potential differences according to any prior experience, the results from wave 2 were stratified accordingly: Students with previous experience working in the PHS in Germany were more likely to describe the PHS as interesting, challenging, and exciting (37%) than students without (26%). Otherwise, we found no relevant differences according to prior PHS experience. More detailed information can be found in Table A6 in Appendix A.

### 3.8. Expectations for Working Life and Career

In order to identify factors that could improve the attractiveness of the PHS as a prospective employer in general, we surveyed criteria that students consider to be particularly important for their future working life in wave 1. A total of 20 different aspects were queried using a five-point Likert scale ranging from “unimportant” to “very important”.

Compatibility of career and family (70%) and good work-life balance (67%) were by far the most important rated aspects for both, medical students and PH&ONM students with PHS interest (Table 5). While medical students with PHS interest ranked direct contact with patients or clients (44%), followed by the opportunity to help people directly (42%) and job security (39%) among the top five aspects of a good work life, PH&ONM students with PHS interest prioritized job security (38%), regular working hours (38%) and good social benefits (34%). Contrary to our expectations, a mere of 13% of all participants with PHS interest considered a high salary to be of particular relevance (12% of all medical students with PHS interest and 15% of all PH&ONM students).

We validated the results from wave 1 by asking all students in wave 2 to rank in total eleven thematically clustered aspects based on their importance regarding their future working life. Again, work-life balance and an appreciative and good working atmosphere were rated highest by all participants. Relevant differences were also found regarding the importance of job security, which was ranked more often as important by students with PHS interest (24%) than by students without (16%). Furthermore, job security was considered as very important more often by PH&ONM students than by medical students. As in wave 1, students without PHS interest considered individual patient care/client-related work more often as important (32%) than students without PHS interest (16%), with the high interest primarily attributable to medical students without PHS interest (41%). More details can be found in Table A7 in the Appendix A.

### 3.9. Expectations for Training and Education

Finally, wishes and expectations for (future) education and training of public health-relevant content were captured in both waves 1 and 2, using various multiple-choice questions. In wave 1, almost twice as many medical students with PHS interest (62%) could imagine learning about public health topics as part of a compulsory elective (in German: Wahlpflichtfach), compared to medical students without interest (34%). Medical students with an interest in the PHS were more likely to envision themselves completing a one-month internship in medical school (in German: Famulatur) or a term of their practical year (in German: Praktisches Jahr) within the PHS than medical students without an interest in the PHS (Appendix A: Table A8).

The public health specialist training, a residency training program qualifying physicians explicitly for the work in the PHS in Germany, was known by 40% of all medical students, with relevant differences between medical students with PHS interest (46%) and those without (38%). When asked what medical specialist training would be of general consideration, a specialty in public health (in German: Facharzt für Oeffentliches Gesundheitswesen) was by far the least frequently mentioned specialty with 6% of all responses in wave 1. In the second survey in wave 2, about 16% of medical students indicated a general interest in the public health specialty (Appendix A: Table A9). Furthermore, PH&ONM students were asked in wave 2 whether they would consider a public health specialist training if such a program was associated with appropriate job and career opportunities and were open to qualified individuals without a medical degree. The vast majority of all PH&ONM students could envision themselves doing such a training (70%).

## 4. Discussion

To the best of our knowledge this is the first empirical study that investigated the attractiveness of the PHS as a potential employer among medical and PH&ONM students in Germany.

**Key finding 1**. *High levels of interest in working in the PHS exist in PH&ONM students—reflecting on relevant activities within the PHS could help to make use of an underutilized pool of qualified employees.*

In line with previous studies [36,57], we found low levels of interest in the PHS among medical students. Almost three out of four medical students expressed a tendentious or categorical lack of interest and only 6% said they could imagine working in the PHS. Our study adds that among those medical students with PHS interest, almost nine out of ten stated that their main interest lay in clinical/client-related activity. In contrast to the medical students, the interest in working in the PHS among PH&ONM students was relatively high, with almost three out of four students expressing a tendentious or definite PHS interest.

In order to ensure the functioning of the PHS with its complex tasks, roles and responsibilities, and to be able to respond instantaneously and efficiently in times of crises, an efficient PHS workforce requires well-qualified professionals possessing comprehensive skills and competencies [58] from a broad variety of disciplines and professions. However, the debate about the shortage of personnel in the PHS in Germany so far has largely focused on medical students and physicians [59], whose basic training does not encompass all the competencies needed for the work in the PHS [60]. While medical professionals are and will remain essential for an effective PHS [59,60,61,62,63], our finding on the disproportionately higher level of interest in the PHS among PH&ONM students indicates that part of the current PHS workforce shortage in Germany could be overcome if the role of public health professionals without medical degrees is strengthened. One solution could be to apply the taxonomy proposed by WHO and the Association of Schools of Public Health in the European Region (APSHER) in their Roadmap for Professionalizing the Public Health Workforce: Instead of primarily focusing on individual professions, they suggest to differentiate by (core) areas of activity and corresponding competencies [61], which in turn could be assigned to professionals with suitable expertise in the particular topic or task. However, establishing such an approach in Germany would first require mapping the numerous activity profiles within the PHS, to provide the basis for an evidence-informed restructuring. While surely a challenging endeavor, focusing on expertise rather than individual professions could help to reach the full potential of a multi- and interdisciplinary public health workforce while balancing workforce supply and employer demand [60,61].

**Key finding 2**. *Students tend to underestimate the impact of public health approaches—overcoming this may increase awareness of the PHS*

Within our logic model, we postulated that a lack of interest in (Figure 1: 1.1) and appreciation of (Figure 1: 1.2) the importance of population-based public health approaches could contribute to low attractiveness of the PHS among students. We found that only one in five participants was able to correctly answer at least three out of the five multiple-choice questions on trends and differences in LE. When asked about the main reasons for the increase in LE over the past 120 years, we found most participants overestimating the importance of medical practices and innovations, while underestimating the importance of changes in living conditions and public health interventions. While we did not find relevant differences in these knowledge domains between individuals with and without PHS interest, we found that considerably more students without interest associated the PHS with a lack of impact.

We are aware that—despite the importance of LE as an indicator to monitor population health [64,65]—knowledge of differences and changes in LE can only serve as a rough proxy for the level of public health knowledge. However, our findings are in line with other studies showing that the contribution of medical care to increased LE in the general population is often overestimated to the detriment of public health measures and improved social conditions, which in turn tends to be underestimated [66].

Underestimating the importance of public health measures for improving health and wellbeing [67] while overestimating the importance of medical interventions may lead individuals who are strongly motivated to make a difference not to pursue a career in public health. There are many factors that could explain why students struggle to assess trends and differences in LE correctly. In addition to gaps in general education and misreporting in the media, the correct assessment is also likely to be hampered by the lack of curricular anchoring within medical studies [68,69]. While our findings indicate a more complex relationship between public health knowledge and interest in working in the PHS, the identified gaps in knowledge support previous calls for including or strengthening public and global health components in the curriculum of all PHS-relevant professions [70]. Integrating PHS-relevant topics into the curricula and highlighting the importance of population-based approaches could raise awareness about the PHS as an attractive employer.


**Key finding 3.**
*Students lack awareness about topics, tasks and activities of the PHS in Germany—more presence in the study programs, tailored training approaches and increased visibility of the PHS could change this*


A second dimension of our logic model assumed that low levels of knowledge about the existence and activities of the PHS (Figure 1: 2.1) may contribute to a lack of interest in working in the PHS among students and young professionals. This is reflected in our findings, as—in addition to the underestimated impact of population-based approaches—the majority of students was not very familiar with the broad spectrum of PHS tasks and activities. We found that many participants did not associate the PHS with parts of its core topics and functions: While only one in six participants did not consider health protection and infection disease prevention to be a core function of the PHS in wave 1, this was the case by one in four participants for health planning and policy advice, behavioral and primordial prevention, and outbreak and crisis management. Primary and secondary prevention as well as support of vulnerable groups was not associated with the PHS by one in three participants each. Not ascribing one of these functions to the PHS was associated with an overall lack of interest in the PHS as a prospective employer among medical students but not among PH&ONM students.

Furthermore, we found that participants stated that many PHS core tasks and activities were not intensively addressed during their studies; with considerable differences between medical students and PH&ONM students, ranging from 61% of the medical students stating (medical) primary prevention and secondary prevention were intensively studied, while only 2% made this statement about health planning and policy advice and 3% about outbreak and crisis management.

Thus, our findings are in line with experts and stakeholders from the public health community calling for a stronger anchoring of the PHS and PHS-related topics in the curricula [68]. Insufficient coverage of public health-relevant topics may contribute to a lack of awareness of the importance of organized efforts needed to improve population health [71]. The PHS here has an essential role to play, above all the LHA which are predestined for adaptation of (complex) public health measures and concepts to local needs due to their municipal anchoring [72]. However, this requires qualified public health professionals who have been trained to deal with the vast amount of wicked problems in public health [61]. To complicate matters, these problems rarely fall within the purview of a single institution or organization [73] and require inter- or even transdisciplinary collaborations among a broad range of professions [74].

In order to equip students and (future) public health professionals with appropriate skills and competencies, existing education and training structures need to be reconsidered. Skills for adopting system-wide approaches are just as relevant as critical public health competencies [75], competencies regarding law and policy formulation and evidence-informed health planning as well as a fundamental understanding of administrative processes and management skills [76,77]; all of them require a direct link between theory and practice [77,78].

In this context, the possibilities of practice-oriented post-graduate training structures should be explored. With the public health specialist training for physicians [79], accompanied by the Academies of Public Health, or the Field Epidemiology Training Program (FETP) of the European Center for Disease Control and the Robert Koch Institute as training site in Germany [80], there are at least two examples of well-established programs in Germany. However, both are either subject-specific (FETP) or profession-specific. Within the scope of the OeGD-Studisurvey we found the broad majority of non-medical students to be interested in a public health specialist training if such programs were associated with appropriate job and career opportunities and open to qualified individuals without a medical degree. This interest could be an opportunity to include students of non-medical study programs in programs preparing for the work in the PHS, especially the work in the LHA. A good example of this is the interdisciplinary training approach of public health consultants and specialists offered by the National Health Service (NHS), which is equally open to medical professionals as well as public health students and students from other PHS-relevant study programs [81].

The measures initiated a few years ago to increase the attractiveness of general practice as a medical specialty in Germany could provide another useful guidance: In addition to strengthening general medicine by implementing the opportunity to perform a corresponding term of the practical year, the specialty also became an integral part of medical training through the establishment of chairs for general practice and family medicine throughout Germany [82]. Analogously to the current developments in the PHS, an amendment to the medical licensing regulations was also initial for general medicine, aiming for a stronger thematic anchoring in the medical curricula. Back in 2016, the establishment of PHS chairs, until then already repeatedly demanded by the public health community [37,39,83], was given political endorsement with a resolution of the Conference of Ministers of Health [3] to strengthen PHS-relevant research and to implement PHS-relevant topics in medical education and training. However, until today PHS chairs are still far from being implemented across the nation. There are so-called “linked professorships” (in German: Brückenprofessuren) at the federal level, which are well suited for the transfer of scientific findings into (local) practice and vice versa due to their double appointment in science and practice [70,84]. Without a comprehensive implementation of regular professorships and chairs of PHS, however, it will be difficult to integrate PHS-relevant topics into the (medical) curriculum on a regular basis. The PHS professorships at the universities of Cologne and Frankfurt am Main, which have been advertised in the summer of 2022, are an important first step in this regard.


**Key finding 4.**
*Medical and PH&ONM students are interested in core topics and functions of the PHS in Germany—expanding on and emphasizing them could increase attractiveness*


Another assumed barrier according to our logic model was that students might just not be interested in core topics and functions of the PHS and the resulting tasks and activities that working in the PHS would involve (Figure 1: 2.2).

This is reflected in our data, as we found that individuals who stated they were interested in working on or in one of these core topics and functions were more likely to consider the PHS as a potential career path. However, we found that both medical students and PH&ONM students were generally interested in selected topics and areas: Even among those individuals who indicated that they were not interest in working in the PHS, between one in four and two in three participants stated, that they could envision working in at least one of the listed core activities.

These findings indicate that a general interest in the core topics and functions of the PHS exists, although this does not necessarily translate into a general PHS interest. However, our findings could be supportive in increasing the attractiveness of the PHS: for example, strengthening topics and functions considered as attractive by many students (e.g., prevention and health promotion) could lead to the PHS be perceived as more attractive. Similar suggestions have been made by other experts and stakeholders in the past [20,59]. Furthermore, emphasizing these themes and functions in public awareness campaigns could also serve as a valuable strategy.


**Key finding 5:**
*The PHS in Germany has an image problem, which needs to be addressed—Existing positive associations could form the core of a new public image*


The final dimension of our logic model addressed how the PHS is perceived among students by focusing on barriers hindering individuals in striving for a career in the PHS in Germany. Here, we distinguished between a negative perception of the PHS due to unjustified stereotypes, which e.g., could be overcome by factual knowledge and experience (Figure 1: 3.1), and a lack of attractiveness of known and experienced characteristics of the PHS, which could not be overcome through knowledge and experience as they require fundamental changes within the PHS (Figure 1: 3.2).

As our study primarily addressed students with limited insights into the PHS as well as due to our cross-sectional methodology, we are not able to clearly distinguish between the two theses. Nonetheless, our results provide important qualitative and quantitative findings that provide initial empirical insights. While the findings on image and perception of the PHS as a potential employer, derived from an in-depth qualitative analysis, are discussed in more detail in the accompanying paper of the OeGD-Studisurvey [48], the findings of the quantitative analysis presented in this paper emphasizes the importance of the public image and perception of the PHS.

We found considerably more participants who could imagine working in the PHS having positive associations and attitudes towards the PHS, as did those who were not interested in working there. This includes the PHS being associated with offering interesting, diverse, and exciting work, the tasks within it being challenging or ambitious, resulting in a high impact, or being modern and innovative (or at least not traditional and outdated).

These positive aspects could be utilized by emphasizing them in public awareness campaigns and by engaging in reforms to further strengthen this part of the PHS employer profile. Furthermore, they could be combined with other messages reflective of values important to students who stated that they could imagine working for the PHS, such as that it would provide an appreciative and good working atmosphere, offer a good work-life balance, and that the work profile consists of a diverse set of activities and allows for creative freedoms. Shaping a new public image of the PHS in this way may help to attract young professionals who currently strive for other career paths.

However, it needs to be acknowledged that the PHS has an image problem: Even among those participants who expressed an interest in working in the PHS, one in three medical students considered working in the PHS as boring and monotonous, one in four medical students as not challenging and not ambitious, every second PH&ONM student considered the PHS as outdated and traditional, and nine out of ten participants with interest in the PHS considered the PHS as bureaucratic. While it is beyond the scope of this publication to distinguish between unjustified stereotypes and experienced negative characteristics of the PHS due to existing underlying problems and deficits, it should be noted that changing the public image alone will not lead to a sustainable increase in the attractiveness of the PHS. To do so, the underlying factual problems and deficits must be addressed in the context of an appropriate structural reform [85,86].

In doing so, experiences from additional good examples and approaches should serve as a basis: The results of the OeGD-Studysurvey could serve as a starting point for the development of a continuous monitoring of the public health workforce along the lines of the PHWINS study [87], just as they should be taken into account in the development of new qualification models, as is currently being done in Germany, for example, in the EvidenzOEGD study [88] or at the European level in the SEEEPHI study [89]. This requires a joint discourse between science, politics and the professional public, which the present results are intended to encourage.

## 5. Strength and Limitations

The OeGD-Studisurvey has several strengths and limitations. Although the data collection is based on a comprehensive recruitment strategy, it is not a randomly selected sample. However, we found that, particularly in wave 1, the population of medical students is comparable to the population of a large survey of more than 13,000 medical students in Germany conducted in 2018 [57]. Similarities can be seen, for example, with regard to interest in working in the PHS or interest in a medical specialist qualification. Unfortunately, for the PH&ONM students, no such reference exists, and it cannot be ruled out that individuals with interest in the PHS are overrepresented due to self-selection.

The proportion of students with a general interest in PHS is higher in wave 2 than in wave 1. There are potentially different explanations for this which must be considered when interpreting the data: We assume that the supposed increase in the attractiveness of the PHS among students overall is mainly due to the slight shift in the population toward more PHS-interested students and fewer medical students without general PHS interest in wave 2. However, due to the cross-sectional design, no causal conclusions can be drawn here. The study design aimed to describe interest in working in the PHS at two different time points. Since the first survey wave was not designed as a follow-up, the two samples do not include the same participants. Thus, the OeGD-Studisurvey can provide important indications, but it is not capable of explaining underlying causes.

It also has to be considered that the population surveyed are students, whose perception and attitudes might change in the early phase of their professional career. Here, conducting additional research among professionals in early stages of their career within and outside of the PHS might provide valuable insights. 

The OeGD-Studisurvey was conducted before and in the early phases of the COVID-19 pandemic. During the pandemic, the PHS in Germany has received high levels of media, political, and public attention. This has likely raised awareness about the PHS among students and young professionals and may have sharpened their perception of it, both in a favorable or less favorable way. Additional research—for example in the form of a third wave of the OeGD-Studisurvey or even in the context of a continuous monitoring analogous to the PH WINS study in the United States [87]—might be a possible solution to better assess the development of perceptions of the PHS among students and young professionals in the future.

Despite these limitations, this survey is—as far as we know—the most comprehensive empirical study to date on the perceived attractiveness of the PHS in Germany among students. The general lack of empirical evidence on this topic further highlights the importance of this study.

## 6. Conclusions

In the wake of the COVID-19 pandemic, the PHS has received a great deal of political attention. Existing deficits were discussed publicly for the first time and initial steps to modernize existing structures have already been taken: for example, with the Pact for the Public Health Service in Germany (in German: OeGD-Pakt) [90] numerous new permanent job positions were created. Medical students meanwhile have the opportunity to conduct part of their electives in the PHS, and the upcoming PHS professorships can help anchoring PHS-relevant topics in the (medical) studies. All of these developments represent a unique opportunity for engaging in a process to reform the PHS towards increasing its attractiveness to sustainably improve recruitment and retention of qualified employees for the PHS in the future.

We believe that such a reform process should be guided by an evidence-informed, deliberative process with all stakeholder involved. The results of and suggestions derived from the qualitative and quantitative analysis of the OeGD-Studisurvey may provide a valuable starting point for such deliberation, as it provides the first empirical data on the perception of the PHS in Germany as a potential employer and the reasons underlying differences in interest among medical and PH & ONM students:

First, in line with established knowledge, our findings showed that while the vast majority clearly had no interest in working in the PHS, a smaller group of students explicitly indicated their interest. A focus on those students and young professionals who are already inclined towards working in the PHS is likely a successful and efficient recruitment strategy. In particular, the high levels of interest among PH&ONM students offers an opportunity to counteract the serious staff shortage. One approach to do so could be to focus recruitment of staff on expertise regarding specific themes, tasks, or skills rather than focusing on degrees or professional backgrounds. This could also help to realize the full potential of a multi- and interdisciplinary PHS workforce, which is urgently needed to address the complex challenges we are currently facing.

Secondly, we found that even students interested in the PHS showed a substantial lack of knowledge about public health and that most students underestimated the importance of public health interventions to improve population health. These misconceptions could lead students interested in achieving impact through their work to seek a career outside of the PHS. The described lack of awareness about the specific topics, tasks and activities of the PHS in Germany among students, could also pose a barrier for entry into the PHS. As also suggested by the German National Academy of Sciences Leopoldina [91], teaching on public health in general and the PHS in particular should be strengthened—not only in the medical studies in Germany, but also in other study programs of particular relevance for improving public health, such as political sciences, economics, or the social sciences.

Thirdly, the PHS is—at least in theory—the central actor when it comes to improving the health of the population and reducing health inequalities. We found that medical and PH&ONM students were interested in the various core topics and functions of the PHS in Germany and especially on the topic and function of prevention, however, many do not know that those activities are even part of the job profile. This indicates that the PHS has so far not yet been sufficiently successful in focusing on precisely these original activities and promoting them accordingly. One approach to address this could be to focus on those core topics and themes of the PHS, which are of particular interest to students, and emphasize those in public awareness campaigns or other situations in which students are exposed to the PHS. A second approach, which may require more structural reform, could be to strengthen the importance of tasks, topics, and themes of particular interest to students—such as prevention and health promotion—within the occupational profile of the PHS.

Furthermore, our findings show that a number of positive associations with the PHS exist, such as that it provides a workplace that is endowed with numerous aspects considered positive and important by students. Emphasizing such associations in public awareness campaigns and utilizing them to strengthen the PHS employer profile can be a valuable resource. However, given that even participants with a strong PHS interest perceive the PHS as bureaucratic, outdated, and the work as boring, monotonous and not challenging, it must be acknowledged that the PHS has an image problem that needs to be addressed and which likely cannot be overcome without fundamental reform processes within. In line with the findings, potential goals maybe to streamline overly bureaucratic processes, to expand automation and digitalization in administrative tasks, to lower rigid hierarchies, or to provide more creative freedoms.

## Figures and Tables

**Table 1:** Characteristics of the study participants (*n* = 3019) in wave 1 and 2 and separated for medical students (*n* = 2058) and public health and other non-medical (PH&ONM) students (*n* = 961) according to their interest in working in the PHS, OeGD-Studisurvey.

	Total	Wave 1 (*n* = 2450)						Wave 2 (*n* = 569)					
		Medical Students			PH&ONM Students			Medical Students			PH&ONM Students		
		Interest	No Interest	*p*-Value *	Interest	No Interest	*p*-Value *	Interest	No Interest	*p*-Value *	Interest	No Interest	*p*-Value *
**Gender**													
male	774 (25.7%)	123 (26.2%)	389 (31.2%)	0.0526	61 (16.1%)	36 (18.5%)	0.6593	24 (32.4%)	46 (36.2%)	0.6465	51 (19.2%)	7 (11.3%)	0.3477
female	2235 (74.1%)	345 (73.4%)	855 (68.6%)		316 (83.6%)	159 (81.5%)		50 (67.6%)	81 (63.8%)		213 (80.4%)	55 (88.7%)	
divers	7 (0.2%)	2 (0.4%)	2 (0.2%)		1 (0.3%)	0 (0%)		0 (0%)	0 (0%)		1 (0.4%)	0 (0%)	
total	3019 (100.0%)	470 (27.4%)	1246 (72.6%)		378 (66.0%)	195 (34.0%)		74 (36.8%)	127 (63.2%)		265 (81.0%)	62 (19.0%)	
**Age in categories**													
≤20	456 (15.7%)	74 (15.8%)	224 (18.2%)	0.1281	30 (8.0%)	34 (17.5%)	0.0001	6 (9.0%)	24 (22.2%)	0.0542	17 (7.6%)	9 (16.7%)	0.2131
21–25	1467 (50.4%)	249 (53.2%)	680 (55.3%)		148 (39.4%)	91 (46.9%)		41 (61.2%)	59 (54.6%)		89 (39.6%)	21 (38.9%)	
26–30	647 (22.2%)	104 (22.2%)	251 (20.4%)		103 (27.4%)	39 (20.1%)		13 (19.4%)	21 (19.4%)		60 (26.7%)	12 (22.2%)	
>30	341 (11.7%)	41 (8.8%)	74 (6.0%)		95 (25.3%)	30 (15.5%)		7 (10.4%)	4 (3.7%)		59 (26.2%)	12 (22.2%)	
total	3019 (100.0%)	468 (27.6%)	1229 (72.4%)		376 (66.0%)	194 (34.0%)		67 (38.3%)	108 (61.7%)		225 (80.6%)	54 (19.4%)	
**Next degree**													
bachelor ^1^	374 (12.4%)	0 (0%)	1 (0.1%)	0.0779	149 (39.3%)	73 (37.4%)	0.0177	1 (1.4%)	0 (0%)	0.2044	101 (39.0%)	22 (35.5%)	0.2624
master	412 (13.7%)	2 (0.4%)	5 (0.4%)		176 (46.4%)	74 (37.9%)		1 (1.4%)	0 (0%)		113 (43.6%)	23 (37.1%)	
state exam	2157 (71.7%)	461 (98.1%)	1235 (99.1%)		46 (12.1%)	40 (20.5%)		65 (90.3%)	122 (95.3%)		27 (10.4%)	12 (19.4%)	
PhD or MD	66 (2.2%)	7 (1.5%)	5 (0.4%)		8 (2.1%)	8 (4.1%)		5 (6.9%)	6 (4.7%)		18 (6.9%)	5 (8.1%)	
total	3019 (100.0%)	470 (27.4%)	1246 (72.6%)		379 (66.0%)	195 (34.0%)		72 (36.0%)	128 (64.0%)		259 (80.7%)	62 (19.3%)	
**Second degree**													
yes	514 (17.2%)	40 (8.5%)	91 (7.3%)	0.4010	161 (43.3%)	65 (34.4%)	0.0425	7 (9.3%)	7 (5.5%)	0.2943	93 (36.6%)	24 (39.3%)	0.6919
no	2476 (82.8%)	430 (91.5%)	1155 (92.7%)		211 (56.7%)	124 (65.6%)		68 (90.7%)	121 (94.5%)		161 (63.4%)	37 (60.7%)	
total	3019 (100.0%)	470 (27.4%)	1246 (72.6%)		372 (66.3%)	189 (33.7%)		75 (36.9%)	128 (63.1%)		254 (80.6%)	61 (19.4%)	
**Experiences in the PHS in Germany** (assessed only wave 2)													
yes	201 (35.3%)							28 (37.3%)	30 (23.4%)	0.0344	115 (43.4%)	20 (32.3%)	0.1088
no	368 (64.7%)							47 (62.7%)	98 (76.6%)		150 (56.6%)	42 (67.7%)	
total	569 (100.0%)							75 (36.9%)	128 (63.1%)		265 (81.0%)	62 (19.0%)	

* In group-comparison between students with and without interest in working in the Public Health Service (PHS) using Chi^2^-test (Fisher’s exact test for ε_ij_ < 5). For reasons of readability, missing values are not shown separately. Abbreviations: PhD = Doctor of philosophy, MD = Medical doctor. ^1^ Although, we did not contact the secretariats of bachelor’s degree programs, it was possible for students with a bachelor’s degree to participate in the survey. An indication of the bachelor’s degree was possible, for example, if persons are enrolled in more than one study program (second degree) and next obtain a bachelor’s degree.

**Table 2:** General interest in working in the Public Health Service (PHS) among students in Germany by territorial authority and separated for wave 1 (*n* = 2450) and 2 (*n* = 569), OeGD-Studisurvey.

	Total	Wave 1			Wave 2		
		Medical Students	PH&ONM Students	*p*-Value *	Medical Students	PH&ONM Students	*p*-Value *
**PHS in general**							
yes	376 (13%)	104 (6%)	151 (26%)	<0.001	9 (4%)	112 (34%)	<0.001
rather yes	813 (29%)	366 (21%)	228 (40%)		66 (33%)	153 (47%)	
rather no	1121 (40%)	810 (47%)	143 (25%)		113 (56%)	55 (17%)	
no	510 (18%)	436 (25%)	52 (9%)		15 (7%)	7 (2%)	
**PHS on local level**							
yes	273 (10%)	54 (3%)	126 (22%)	<0.001	9 (5%)	84 (26%)	<0.001
rather yes	567 (20%)	230 (13%)	164 (28%)		48 (24%)	125 (39%)	
rather no	1145 (40%)	761 (43%)	188 (32%)		109 (54%)	87 (27%)	
no	870 (30%)	708 (40%)	102 (18%)		35 (17%)	25 (8%)	
**PHS on federal or national level**							
yes	452 (16%)	117 (7%)	213 (36%)	<0.001	15 (8%)	107 (33%)	<0.001
rather yes	812 (28%)	392 (22%)	213 (36%)		83 (42%)	124 (38%)	
rather no	951 (33%)	709 (40%)	94 (16%)		78 (39%)	70 (22%)	
no	658 (23%)	547 (31%)	64 (11%)		24 (12%)	23 (7%)	
**PHS interest** *(binary index* ^1^ *)*							
yes	1189 (42%)	470 (27%)	379 (66%)	<0.001	75 (37%)	265 (81%)	<0.001

* In group-comparison between medical and PH&ONM students using Chi^2^-test (Fisher’s exact test for ε_ij_ < 5). ^1^ The binary index PHS interest is defined through the responses “yes” or “rather yes” to the question on general interest in working in the PHS in Germany.

**Table 3:** Specific knowledge about and interest in topics, tasks and activities of the PHS in Germany: Absolute and relative proportion of core public health fields of activities regarding their PHS attribution, coverage within the study curricula, and consideration of prospective employment by field of study and PHS interest in wave 1, OeGD-Studisurvey.

	Total			Medical Students			PH&ONM Students		
	Medical Students	PH&ONM Students	*p*-Value *	Interest	No Interest	*p*-Value **	Interest	No Interest	*p*-Value **
**Core public health fields of activities that are not counted as part of the PHS** *(In your opinion, which of the fields of activity belong to the area of responsibility of the public health service (PHS)?)*									
Health protection and infection disease prevention	295 (17.3%)	82 (14.5%)	0.1265	56 (12.8%)	218 (18.7%)	0.0050	50 (13.6%)	30 (17.4%)	0.2452
Outbreak and crisis management	379 (22.2%)	157 (28.0%)	0.0045	94 (21.5%)	254 (21.6%)	0.9567	105 (29.2%)	46 (26.6%)	0.5364
(Medical) Primary prevention and secondary prevention	583 (34.1%)	182 (32.4%)	0.4674	128 (29.2%)	414 (35.2%)	0.0220	109 (29.9%)	60 (34.7%)	0.2693
Support of vulnerable groups	589 (34.6%)	170 (30.4%)	0.0647	123 (28.3%)	437 (37.3%)	0.0008	101 (27.7%)	57 (33.1%)	0.2012
Health planning and policy advice	445 (26.1%)	155 (27.3%)	0.5827	109 (24.9%)	313 (26.8%)	0.4435	92 (25.1%)	54 (30.7%)	0.1673
Behavioral and primordial prevention	479 (28.1%)	170 (30.2%)	0.3466	107 (24.5%)	345 (29.5%)	0.0500	106 (29.0%)	53 (30.8%)	0.6606
**Core public health fields of activities that were intensively covered in the study curriculum** * (How intensively are or were the following aspects addressed during your main studies?)*									
Health protection and infection disease prevention	649 (42.2%)	172 (30.8%)	<0.0001	143 (36.0%)	467 (44.3%)	0.0046	118 (32.9%)	46 (26.4%)	0.1314
Outbreak and crisis management	39 (2.9%)	35 (6.9%)	0.0001	10 (2.9%)	26 (2.8%)	0.9248	23 (7.0%)	9 (5.8%)	0.6184
(Medical) Primary prevention and secondary prevention	992 (61.3%)	295 (52.4%)	0.0002	236 (56.5%)	695 (62.7%)	0.0252	214 (59.4%)	70 (40.2%)	<0.0001
Support of vulnerable groups	192 (13.2%)	240 (43.9%)	<0.0001	41 (11.1%)	134 (13.2%)	0.3092	173 (49.1%)	55 (32.0%)	0.0002
Health planning and policy advice	27 (2.0%)	167 (30.7%)	<0.0001	9 (2.6%)	16 (1.7%)	0.3011	133 (37.8%)	30 (17.9%)	<0.0001
Behavioral and primordial prevention	516 (33.5%)	299 (53.4%)	<0.0001	129 (32.4%)	358 (34.0%)	0.5759	217 (59.6%)	71 (41.5%)	0.0001
**Core public health fields of activities that can be considered for employment after graduation** *(Are the following occupational fields a possibility for you after your studies?)*									
Health protection and infection disease prevention	845 (46.9%)	291 (49.1%)	0.3681	307 (66.9%)	481 (39.0%)	<0.0001	213 (57.6%)	69 (35.8%)	<0.0001
Outbreak and crisis management	625 (34.7%)	199 (33.7%)	0.6534	215 (46.7%)	372 (30.2%)	<0.0001	141 (38.1%)	50 (26.6%)	0.0067
(Medical) Primary prevention and secondary prevention	1233 (68.7%)	315 (53.5%)	<0.0001	371 (81.2%)	776 (63.2%)	<0.0001	228 (62.0%)	76 (40.0%)	<0.0001
Support of vulnerable groups	1017 (56.8%)	375 (64.5%)	0.0010	321 (70.4%)	630 (51.4%)	<0.0001	264 (72.1%)	95 (50.8%)	<0.0001
Health planning and policy advice	594 (33.0%)	356 (60.1%)	<0.0001	238 (51.5%)	314 (25.6%)	<0.0001	255 (68.7%)	88 (46.6%)	<0.0001
Behavioral and primordial prevention	865 (48.2%)	385 (65.0%)	<0.0001	288 (62.7%)	520 (42.4%)	<0.0001	268 (72.2%)	95 (49.5%)	<0.0001

* Comparison between medical and PH&ONM students ** In group-comparison between medical and PH&ONM students with and without PHS interest using Chi^2^-test (Fisher’s exact test for ε_ij_ < 5).

**Table 4:** Interest in core public health fields of activities regarding their coverage during the main studies and attribution to the PHS: Absolute and relative proportions separated by medical and PH&ONM students according to their PHS interest in wave 1 of the OeGD-Studisurvey.

	Total	Medical Students			PH&ONM Students		
		Interest	No Interest	*p*-Value *	Interest	No Interest	*p*-Value *
*Core public health fields of activity are considered as interesting and is/was intensively covered during the main studies* **Interest in core public health fields of activity by coverage during the main studies**							
Health protection, NCD prevention	474 (47.0%)	106 (40.3%)	232 (54.0%)	0.0005	85 (41.7%)	25 (39.7%)	0.7797
Outbreak and crisis management	42 (6.6%)	6 (3.8%)	11 (3.9%)	0.9785	14 (11.4%)	7 (15.9%)	0.4370
(Med.) Primary & sec. prevention	937 (67.3%)	206 (61.9%)	486 (69.9%)	0.0099	148 (68.5%)	41 (60.3%)	0.2100
Support of vulnerable groups	314 (26.6%)	28 (10.9%)	78 (14.9%)	0.1237	146 (57.5%)	43 (47.8%)	0.1119
Health planning, policy advice	149 (19.0%)	6 (3.2%)	9 (3.8%)	0.7274	106 (43.3%)	23 (28.4%)	0.0177
Behavioral, primordial prevention	536 (47.7%)	99 (39.4%)	188 (41.1%)	0.6602	174 (66.7%)	48 (53.9%)	0.0312
*Core public health fields of activity are considered as interesting and belong to the responsibility of the PHS* **Interest in core public health fields of activity by PHS attribution**							
Health protection, NCD prevention	918 (85.2%)	256 (87.7%)	383 (83.4%)	0.1127	185 (88.1%)	50 (82.0%)	0.2145
Outbreak and crisis management	595 (75.6%)	162 (79.8%)	274 (76.1%)	0.3142	104 (74.8%)	27 (61.4%)	0.0846
(Med.) Primary & sec. prevention	969 (66.0%)	248 (70.1%)	466 (62.7%)	0.0171	166 (74.1%)	47 (70.1%)	0.5211
Support of vulnerable groups	904 (68.8%)	223 (74.3%)	383 (64.0%)	0.0019	191 (73.5%)	59 (66.3%)	0.1953
Health planning, policy advice	684 (75.2%)	175 (78.5%)	222 (74.2%)	0.2629	195 (77.1%)	59 (68.6%)	0.1174
Behavioral, primordial prevention	888 (74.9%)	211 (76.2%)	373 (75.7%)	0.8729	193 (73.1%)	65 (73.9%)	0.8894

* In group-comparison between medical and PH&ONM students with and without PHS interest using Chi^2^-test (Fisher’s exact test for ε_ij_ < 5); NCD = Non-communicable diseases; (Med.) Primary & sec. prevention = (medical) primary and secondary prevention.

**Table 5:** Expectations of future working life and career among medical students and PH&ONM students in Germany according to their PHS interest: Absolut and relative proportions of aspects considered as “very important” in wave 1, OeGD-Studisurvey.

	Total			Medical Students			PH&ONM Students		
	Interest	No Interest	*p*-Value *	Interest	No Interest	*p*-Value **	Interest	No Interest	*p*-Value **
Good work-life balance	570 (67.2%)	875 (60.8%)	0.0061	326 (69.5%)	761 (61.1%)	0.0045	244 (64.4%)	114 (58.8%)	0.3552
Compatibility of career and family	588 (69.7%)	992 (69.0%)	0.4260	338 (72.2%)	861 (69.2%)	0.4193	250 (66.5%)	131 (67.5%)	0.1348
Flexible working hours	234 (27.9%)	260 (18.2%)	<0.0001	118 (25.3%)	197 (16.0%)	<0.0001	116 (31.0%)	63 (32.5%)	0.3765
Regulated working hours without weekend/night duties	263 (31.2%)	283 (19.8%)	<0.0001	122 (26.1%)	214 (17.3%)	<0.0001	141 (37.5%)	69 (35.8%)	0.1409
High salary	109 (12.9%)	199 (13.8%)	0.2706	54 (11.5%)	154 (12.4%)	0.5723	55 (14.7%)	45 (23.2%)	0.0312
Job security	326 (38.5%)	574 (40.0%)	0.7353	183 (39.0%)	506 (40.7%)	0.5282	143 (37.9%)	68 (35.2%)	0.2564
Regularly new content	123 (14.6%)	214 (15.0%)	0.9052	72 (15.5%)	184 (14.9%)	0.8004	51 (13.5%)	30 (15.6%)	0.7538
Realize own projects	232 (27.5%)	350 (24.3%)	0.0825	124 (26.4%)	291 (23.4%)	0.3983	108 (28.7%)	59 (30.3%)	0.9174
Great social benefits	287 (34.1%)	435 (30.4%)	0.0072	161 (34.5%)	375 (30.3%)	0.2341	126 (33.6%)	60 (31.1%)	<0.0001
Being able to help people directly	300 (35.6%)	685 (47.8%)	<0.0001	196 (41.9%)	627 (50.5%)	0.0004	104 (27.7%)	58 (30.2%)	0.1075
Being able to influence general conditions	249 (29.8%)	282 (19.8%)	<0.0001	133 (28.7%)	241 (19.5%)	<0.0001	116 (31.1%)	41 (21.6%)	0.0001
Flat hierarchical structures	193 (23.1%)	315 (22.3%)	0.4468	132 (28.5%)	285 (23.3%)	0.0048	61 (16.4%)	30 (16.2%)	0.3522
Being able to take on responsibility	166 (19.6%)	267 (18.6%)	0.8045	86 (18.3%)	222 (17.9%)	0.5933	80 (21.2%)	45 (23.2%)	0.0976
Career advancement opportunities	220 (26.0%)	388 (27.0%)	0.8074	126 (27.0%)	334 (26.9%)	0.7668	94 (24.9%)	54 (27.7%)	0.7649
Being active in research/science	103 (12.2%)	172 (12.0%)	0.7980	46 (9.8%)	124 (10.0%)	0.7938	57 (15.1%)	48 (24.9%)	0.0168
Possibility to do a doctorate	87 (10.7%)	174 (12.5%)	0.0044	50 (11.2%)	136 (11.3%)	0.7200	37 (10.0%)	38 (20.0%)	0.0016
Working in a team	267 (31.4%)	522 (36.3%)	0.0152	177 (37.7%)	480 (38.6%)	0.2509	90 (23.7%)	42 (21.5%)	0.2898
Direct contact with patients/clients	258 (30.5%)	758 (52.9%)	<0.0001	208 (44.3%)	722 (58.2%)	<0.0001	50 (13.3%)	36 (18.7%)	0.1461
Possibility to work abroad	140 (16.5%)	252 (17.6%)	0.2671	108 (23.1%)	233 (18.8%)	0.1184	32 (8.4%)	19 (9.8%)	0.8566
Working in an inter- and multidisciplinary team	237 (28.1%)	401 (27.9%)	0.5435	175 (37.6%)	368 (29.6%)	0.0059	62 (16.4%)	33 (17.1%)	0.8965

* Comparison between students with and without PHS interest; ** In group-comparison between medical and PH&ONM students using Chi^2^-test (Fisher’s exact test for ε_ij_ < 5).

## Data Availability

After project completion, the quantitative data of the OeGD-Studisurvey will be available on https://osf.io/uxftz. To prevent identification of individuals through the combination of the open-ended answers and socio-demographic data, the unredacted dataset will not be publicly available due to privacy reasons. The data presented in this study are available on request from the corresponding author.

## Supplementary Materials

The following supporting information can be downloaded at: https://www.mdpi.com/article/10.3390/ijerph191811838/s1, File S1: S1_OeGD-Studisurvey_Sampling_procedure; File S2: S2_OeGD-Studisurvey_PartI_Questionaire_wave1; File S3: S3_OeGD-Studisurvey_PartI_Questionaire_wave2.

## Abbreviations

	English	German
AOEGW	Academy of Public Health Services	Akademie für Öffentliches Gesundheitswesen in Düsseldorf
bvmd	German Medical Students’ Association	Bundesvertretung der Medizinstudierenden in Deutschland e.V.
BVOEGD	Federal Association for Physicians in the PHS	Bundesverband für Ärztinnen und Ärzte im Öffentlichen Gesundheitsdienst
HRK	German Rectors’ Conference	Hochschulrektorenkonferenz
IMPP	Institute for Medical and Pharmaceutical Examination Question	Institut für medizinische und pharmazeutische Prüfungsfragen
LHA	Local health authorities	Kommunale Gesundheitsämter
MS	medical students	Medizinstudierende
NOEG	German Network of Young Professionals in Public Health	Nachwuchsnetzwerk Oeffentliche Gesundheit
PH	Public Health	Public Health
PH&ONM	public health and other non-medical students	Public Health Studierende und Studierende anderer nicht-medizinischer Studiengänge
PHS	Public Health Service	Öffentlicher Gesundheitsdienst (in German: OEGD)
PJ	Practical year	Praktisches Jahr (Studienabschnitt im Medizinstudium)
RKI	Robert Koch Institute	Robert Koch-Institut
SDH	Social determinants of health	Soziale Determinanten von Gesundheit

## Appendix A. Additional Tables and Figures

**Table A1:** Preferred fields of activity after graduation: Absolute and relative proportion of the fields of activity with the highest priority by field of study and PHS interest in wave 1, OeGD-Studisurvey.

	Total	Medical Students			PH&ONM Students		
		Interest	No Interest	*p*-Value *	Interest	No Interest	*p*-Value *
clinical/client-related activity	1692 (74.0%)	371 (83.4%)	1085 (92.8%)	<0.001	66 (19.2%)	56 (30.6%)	<0.001
PH activities outside PHS	100 (4.4%)	19 (4.3%)	10 (0.9%)		50 (14.6%)	18 (9.8%)	
PH activities within PHS	143 (6.2%)	20 (4.5%)	3 (0.3%)		107 (31.2%)	10 (5.5%)	
research and academia	181 (7.9%)	15 (3.4%)	33 (2.8%)		72 (21.0%)	47 (25.7%)	
private sector	95 (4.2%)	9 (2.0%)	27 (2.3%)		25 (7.3%)	26 (14.2%)	
international	77 (3.4%)	11 (2.5%)	11 (0.9%)		23 (6.7%)	26 (14.2%)	

* In group-comparison between medical and PH&ONM students using Chi^2^-test (Fisher’s exact test for ε_ij_ < 5); PH = public health.

**Table A2:** Preferred fields of activity after graduation: Absolute and relative proportion of all “Yes, definitely” responses by field of study and PHS interest in wave 2, OeGD-Studisurvey.

	Total	Medical Students			PH&ONM Students		
		Interest	No Interest	*p*-Value *	Interest	No Interest	*p*-Value *
clinical/client-related activity	311 (58%)	37 (50.7%)	91 (72.2%)	0.0032	35 (14.6%)	11 (18.3%)	0.1773
PH activities outside PHS	286 (51%)	7 (9.5%)	2 (1.6%)	<0.0001	77 (29.5%)	11 (17.7%)	<0.0001
PH activities within PHS	382 (68%)	18 (24.3%)	2 (1.6%)	<0.0001	127 (48.5%)	7 (11.3%)	<0.0001
research and academia	314 (56%)	17 (23.0%)	17 (13.5%)	0.2145	74 (28.2%)	13 (21.3%)	0.5459
private sector	175 (31%)	5 (6.8%)	5 (3.9%)	0.0353	37 (14.2%)	8 (12.9%)	0.3924
international	271 (49%)	17 (23.3%)	7 (5.5%)	0.0002	68 (26.3%)	10 (16.1%)	0.0036

* In group-comparison between medical and PH&ONM students using Chi^2^-test (Fisher’s exact test for ε_ij_ < 5); PH = public health.

**Table A3:** General public health knowledge with focus on life expectancy (LE): Absolute and relative proportion of correct answers to estimated differences in LE by field of study and PHS interest in wave 1, OeGD-Studisurvey.

	Total	Medical Students			PH&ONM Students		
		Interest	No Interest	*p*-Value *	Interest	No Interest	*p*-Value *
**Number (and proportion) of correct answers for estimating life expectancy**							
Average shift in LE in rich countries	1828 (74.9%)	351 (74.7%)	935 (75.3%)	0.7774	293 (77.3%)	146 (75.3%)	0.5831
Average shift in LE in poor countries	236 (9.7%)	38 (8.1%)	133 (10.7%)	0.1127	35 (9.3%)	19 (9.7%)	0.8739
Average difference in LE among women with low and high SES	700 (28.8%)	118 (25.2%)	356 (28.7%)	0.1553	123 (32.5%)	57 (29.5%)	0.4646
Average difference in LE among men with low and high SES	274 (11.3%)	55 (11.8%)	146 (11.7%)	0.9971	38 (10.1%)	20 (10.3%)	0.9314
Average difference in LE between women and men	1332 (54.6%)	282 (60.1%)	673 (54.1%)	0.0251	200 (52.9%)	91 (46.7%)	0.1567
**Sumscore of correct answers**							
Almost all correct (4 to 5)	3 (0.1%)	1 (0.2%)	1 (0.1%)	0.4732	1 (0.3%)	0 (0.0%)	1.0000
At least 3 correct answers	531 (22.0%)	101 (21.7%)	277 (22.4%)	0.7323	85 (22.7%)	34 (17.8%)	0.1792
Almost none correct (0 to 1)	893 (36.9%)	170 (36.5%)	452 (36.6%)	0.9549	129 (34.4%)	74 (38.7%)	0.3083

* In group-comparison between medical and PH&ONM students using Chi^2^-test (Fisher’s exact test for ε_ij_ < 5), LE = life expectancy, SES = socio-economic status. Changes within Germany as well as between countries with high and low income per capita were differentiated and differences between men and women were addressed. The average shift in LE in rich countries was answered correctly by the majority of all participants (75%), while less than 10% correctly estimated LE differences in poorer countries.

**Table A4:** Measures with assumed highest impact regarding the increase in life expectancy within the last 120 years: Absolute and relative proportion of the top three ranked measures by field of study and PHS interest in wave 1, OeGD-Studisurvey.

	Total	Medical Students			PH&ONM Students		
		Interest	No Interest	*p*-Value *	Interest	No Interest	*p*-Value *
Medical therapy of infectious diseases	1664 (68.7%)	326 (69.7%)	870 (70.3%)	0.7863	246 (65.4%)	122 (62.9%)	0.5482
Primary medical prevention	1370 (56.6%)	271 (58.0%)	701 (56.8%)	0.6370	181 (48.3%)	124 (63.9%)	0.0004
Poverty reduction, reduction of malnutrition and other measures	1234 (51.0%)	257 (55.0%)	645 (52.2%)	0.2935	174 (46.8%)	91 (47.2%)	0.9323
Medical therapy and prevention of NCDs	1135 (47.1%)	218 (46.9%)	572 (46.4%)	0.8674	187 (50.0%)	95 (49.0%)	0.8157
Changing living environments	1074 (44.5%)	182 (39.3%)	521 (42.2%)	0.2894	199 (53.2%)	89 (46.1%)	0.1093
Secondary medical prevention	412 (17.1%)	70 (15.1%)	218 (17.7%)	0.2021	73 (19.5%)	26 (13.4%)	0.0684
Non-medical prevention and health promotion measures	389 (16.2%)	80 (17.2%)	187 (15.3%)	0.3412	68 (18.1%)	35 (18.0%)	0.9784

* In group-comparison between medical and PH&ONM students using Chi^2^-test (Fisher’s exact test for ε_ij_ < 5), NCDs = Non-communicable diseases.

**Table A5:** Polarity profile: Characteristics associated with an activity in the PHS: Absolute and relative proportion of grouped responses by field of study and PHS interest in wave 1 and wave 2, OeGD-Studisurvey.

	Medical Students			PH&ONM Students		
	Interest	No Interest	*p*-Value *	Interest	No Interest	*p*-Value *
**Interesting vs. boring**						
interesting, diverse, exciting	144 (38.0%)	55 (7.2%)	<0.0001	246 (48.3%)	23 (17.7%)	<0.0001
neutral	104 (27.4%)	157 (20.4%)		155 (30.5%)	41 (31.5%)	
boring, monotonous	131 (34.6%)	557 (72.4%)		108 (21.2%)	66 (50.8%)	
**Challenging vs. not challenging**						
challenging, ambitious	170 (45.0%)	179 (23.3%)	<0.0001	320 (62.9%)	59 (45.4%)	0.0011
neutral	115 (30.4%)	303 (39.5%)		128 (25.1%)	45 (34.6%)	
not challenging, not ambitious	93 (24.6%)	285 (37.2%)		61 (12.0%)	26 (20.0%)	
**With high vs. low impact**						
high impact	297 (78.6%)	534 (69.4%)	0.0032	376 (74.0%)	72 (55.4%)	0.0002
neutral	59 (15.6%)	157 (20.4%)		85 (16.7%)	37 (28.5%)	
low impact	22 (5.8%)	78 (10.1%)		47 (9.3%)	21 (16.2%)	
**Focusing on global vs. local health problems**						
global	104 (27.5%)	181 (23.5%)	0.2347	93 (18.3%)	30 (23.1%)	0.2994
neutral	106 (28.0%)	246 (32.0%)		150 (29.5%)	31 (23.8%)	
local	168 (44.4%)	342 (44.5%)		266 (52.3%)	69 (53.1%)	
**modern vs. outdated**						
modern, innovative	73 (19.3%)	120 (15.6%)	0.0378	83 (16.4%)	11 (8.5%)	0.0596
neutral	153 (40.5%)	280 (36.4%)		157 (31.0%)	40 (30.8%)	
traditional, outdated	152 (40.2%)	369 (48.0%)		267 (52.7%)	79 (60.8%)	
**unbureaucratic vs. bureaucratic**						
unbureaucratic	2 (0.5%)	4 (0.5%)	0.0787	2 (0.4%)	1 (0.8%)	0.4788
neutral	31 (8.2%)	38 (4.9%)		55 (10.8%)	11 (8.4%)	
bureaucratic	345 (91.3%)	726 (94.5%)		450 (88.8%)	119 (90.8%)	
**with vs. without career opportunities**						
lack of career opportunities	62 (16.4%)	123 (16.0%)	0.9801	103 (20.4%)	20 (15.4%)	0.1089
neutral	140 (37.1%)	285 (37.1%)		226 (44.7%)	52 (40.0%)	
lack of career opportunities	175 (46.4%)	360 (46.9%)		177 (35.0%)	58 (44.6%)	
**ambitious vs. relaxed**						
for ambitious people	48 (12.7%)	50 (6.5%)	0.0001	134 (26.3%)	24 (18.5%)	0.0036
neutral	93 (24.6%)	155 (20.2%)		208 (40.9%)	43 (33.1%)	
for those interested in relaxed job	237 (62.7%)	563 (73.3%)		167 (32.8%)	63 (48.5%)	

* In group-comparison between medical and PH&ONM students using Chi^2^-test (Fisher’s exact test for ε_ij_ < 5).

**Table A6:** Polarity profile: Characteristics associated with an activity in the Public Health Services (PHS) by previous experience within the PHS: Absolute and relative proportion of grouped responses by field of study and PHS interest in wave 2, OeGD-Studisurvey.

Previous Experience within the PHS			
	Yes	No	*p*-Value *
**Interesting vs. boring**			
interesting and varied	73 (36.5%)	92 (25.6%)	0.0246
neutral	50 (25.0%)	106 (29.4%)	
boring and monotonous	77 (38.5%)	162 (45.0%)	
**Challenging vs. not challenging**			
challenging and demanding	88 (44.4%)	159 (43.9%)	0.5681
neutral	56 (28.3%)	116 (32.0%)	
easy and not very demanding	54 (27.3%)	87 (24.0%)	
**With high vs. low impact on population health**			
high impact on population health	140 (70.7%)	261 (72.3%)	0.7847
neutral	38 (19.2%)	61 (16.9%)	
little impact on population health	20 (10.1%)	39 (10.8%)	
**Modern vs. outdated**			
modern and innovative	41 (20.6%)	62 (17.2%)	0.5804
neutral	72 (36.2%)	132 (36.6%)	
traditional and outdated	86 (43.2%)	167 (46.3%)	
**With vs. without scope for action**			
with high scope for action, formative	22 (11.1%)	42 (11.7%)	0.3878
neutral	53 (26.6%)	114 (31.8%)	
with low scope for action, official	124 (62.3%)	203 (56.5%)	
**With vs. without career opportunities**			
many career opportunities	2 (1.0%)	2 (0.6%)	0.6308
neutral	11 (5.5%)	25 (6.9%)	
few career opportunities	187 (93.5%)	333 (92.5%)	
**Ambitious vs. relaxed**			
for people with goals and ambitions	41 (20.6%)	68 (19.0%)	0.8738
neutral	76 (38.2%)	143 (39.9%)	
for people looking for a relaxed job	82 (41.2%)	147 (41.1%)	
**With high vs. low work-life-balance**			
with high work-life-balance	37 (18.6%)	56 (15.5%)	0.6197
neutral	60 (30.2%)	117 (32.4%)	
with low work-life-balance	102 (51.3%)	188 (52.1%)	

* Comparison between students with previous experience within the PHS and students without using Chi^2^-test (Fisher’s exact test for ε_ij_ < 5).

**Table A7:** Expectations for future working life and career: Absolute and relative proportion of grouped responses by field of study and PHS interest in wave 2, OeGD-Studisurvey.

	Total			Medical Students			PH&ONM Students		
	Interest	No Interest	*p*-Value *	Interest	No Interest	*p*-Value **	Interest	No Interest	*p*-Value **
Work-life balance	220 (64.7%)	129 (67.9%)	0.6412	50 (66.7%)	87 (68.0%)	0.9630	170 (64.2%)	42 (67.7%)	0.2390
Flexible working hours	101 (29.7%)	43 (22.6%)	0.1775	13 (17.3%)	21 (16.4%)	0.4473	88 (33.2%)	22 (35.5%)	0.9422
High salary	69 (20.3%)	31 (16.3%)	0.0177	14 (18.7%)	19 (14.8%)	0.0879	55 (20.8%)	12 (19.4%)	0.8887
Job security	83 (24.4%)	30 (15.8%)	0.0256	14 (18.7%)	13 (10.2%)	0.1586	69 (26.0%)	17 (27.4%)	0.2699
Social recognition and reputation	13 (3.8%)	6 (3.2%)	0.8456	2 (2.7%)	4 (3.1%)	0.2740	11 (4.2%)	2 (3.2%)	0.8779
Appreciative, good working atmosphere	216 (63.5%)	123 (64.7%)	0.8764	52 (69.3%)	86 (67.2%)	0.8547	164 (61.9%)	37 (59.7%)	0.4073
Varied activities with scope for creativity	119 (35.0%)	68 (35.8%)	0.9619	32 (42.7%)	45 (35.2%)	0.5203	87 (32.8%)	23 (37.1%)	0.5916
Activity with societal impact	88 (25.9%)	40 (21.1%)	0.3966	21 (28.0%)	30 (23.4%)	0.1799	67 (25.3%)	10 (16.1%)	0.2045
Direct contact with clients and patients	51 (15.0%)	60 (31.6%)	<0.0001	14 (18.7%)	52 (40.6%)	0.0038	37 (14.0%)	8 (12.9%)	0.1223
Training opportunities	34 (10.0%)	28 (14.7%)	0.0950	10 (13.3%)	21 (16.4%)	0.5087	24 (9.1%)	7 (11.3%)	0.7011
Research or scientific activity	26 (7.6%)	12 (6.3%)	0.8207	3 (4.0%)	6 (4.7%)	0.9006	23 (8.7%)	6 (9.7%)	0.8872

* Comparison between students with and without PHS interest; ** In group-comparison between medical and PH&ONM students using Chi^2^-test (Fisher’s exact test for ε_ij_ < 5).

**Table A8:** Preferred ways to gain (practical) experience relevant for tasks and activities of the PHS among medical students in Germany: Absolute and relative proportions, taking into account multiple responses in wave 1, OeGD-Studisyurvey.

	Medical Students with PHS Interest	Medical Students without PHS Interest	*p*-Value *
Elective	293 (62.3%)	418 (33.5%)	<0.0001
One-month internship in medical school (in German: Famulatur)	256 (54.5%)	216 (17.3%)	<0.0001
Term of the practical year(in German: Praktisches Jahr)	109 (23.2%)	28 (2.2%)	<0.0001
None of the above	45 (9.6%)	641 (51.4%)	<0.0001

* In group-comparison between medical students with and without PHS interest using Chi^2^-test (Fisher’s exact test for ε_ij_ < 5).

**Table A9:** Medical specialist training programs that are generally considered as options for medical students, according to their PHS interest: Absolute and relative proportions, taking into account multiple responses in waves 1 and 2, OeGD-Studisurvey.

	Wave 1				Wave 2			
	Total	PHS Interest	No PHS Interest	*p*-Value *	Total	PHS Interest	No PHS Interest	*p*-Value *
Internal medicine	666 (35.7%)	158 (33.6%)	469 (37.6%)	0.1227	101 (40.2%)	26 (34.7%)	67 (52.3%)	0.0147
Pediatrics	465 (24.9%)	125 (26.6%)	308 (24.7%)	0.4248	57 (22.7%)	19 (25.3%)	32 (25.0%)	0.9579
General medicine	532 (28.5%)	155 (33.0%)	334 (26.8%)	0.0115	74 (29.5%)	27 (36.0%)	38 (29.7%)	0.3521
Anesthetics	435 (23.3%)	96 (20.4%)	317 (25.4%)	0.0302	63 (25.1%)	19 (25.3%)	40 (31.2%)	0.3702
Surgery	439 (23.6%)	70 (14.9%)	330 (26.5%)	<0.0001	53 (21.1%)	10 (13.3%)	39 (30.5%)	0.0059
Gynecology	313 (16.8%)	75 (16.0%)	212 (17.0%)	0.6008	43 (17.1%)	7 (9.3%)	26 (20.3%)	0.0407
Psychiatry	224 (12.0%)	77 (16.4%)	130 (10.4%)	0.0007	40 (15.9%)	17 (22.7%)	16 (12.5%)	0.0581
Neurology	326 (17.5%)	92 (19.6%)	213 (17.1%)	0.2308	39 (15.5%)	11 (14.7%)	26 (20.3%)	0.3146
Public Health	109 (5.8%)	84 (17.9%)	16 (1.3%)	<0.0001	41 (16.3%)	30 (40.0%)	4 (3.1%)	<0.0001
Other	317 (17.0%)	70 (14.9%)	223 (17.9%)	0.1403	44 (17.5%)	17 (22.7%)	21 (16.4%)	0.2697
Unclear	536 (28.8%)	166 (35.3%)	306 (24.6%)	<0.0001	93 (37.1%)	30 (40.0%)	40 (31.2%)	0.2055
None	27 (1.4%)	11 (2.3%)	10 (0.8%)	0.0098	6 (2.4%)	1 (1.3%)	2 (1.6%)	1

Percentage of cases takes into account multiple responses and refers to the total of all persons who answered the questions; * In group-comparison between medical and PH&ONM students using Chi^2^-test (Fisher’s exact test for ε_ij_ < 5).
