# Supplementary material for: What Factors Influence the Interest in Working in the Public Health Service in Germany? Part I of the OeGD-Studisurvey
Source: Int J Environ Res Public Health. 2022 Sep 19;19(18):11838. doi: 10.3390/ijerph191811838 (PMC9517554; doi:10.3390/ijerph191811838)
Supplement: Supplementary file 1 [file ijerph-19-11838-s001.zip › S2_OeGD-Studisurvey_PartI_Questionaire_wave1.pdf]

Herzlich Willkommen!

Wir freuen uns, dass du Dir kurz Zeit für die Umfrage nimmst!

Wir wollen herausfinden, (1) welche **Wünsche und Ansprüche** Mediziner_innen und Gesundheitswissenschaftler_innen an ihre zukünftige Arbeit und Ihre Arbeitgeber haben und (2) was das konkret für **Ausbildungs- und Karrierewege** im Bereich Public Health/öffentliche Gesundheit bedeutet.

Je mehr Personen teilnehmen, desto eher können wir aus den Ergebnissen ableiten, welche Weichenstellungen vorgenommen werden müssen um attraktive Jobs und Arbeitsbedingungen zu schaffen. Zum anderen verleiht uns (und dir) eine große Zahl von Antworten mehr **(politische) Einflussmöglichkeiten**, diese Wünsche und Erwartungen auch in deinem Sinne bei den Arbeitgebern durchzusetzen.

Als Dankeschön für dein Engagement verlosen wir unter allen Teilnehmenden ein Kongressticket inkl. Übernachtungskosten zum nächsten BVÖGD-Kongress in Saarbrücken (23. – 25. April 2020), ein Lenovo-Tablet (tabE10) und drei Power-Banks. Lass uns hierzu einfach deine E-Mail-Adresse am Ende der Befragung zukommen, wenn du an der Verlosung teilnehmen möchtest. Die Verlosung verläuft natürlich unabhängig von der Befragung.

Unsere Befragung ist **anonym**: Wir fragen ein paar wenige personenbezogene Daten ab (z.B. was du studierst), aber du als Individuum bist nicht mehr identifizierbar und Rückschlüsse auf die jeweilige Person sind nicht mehr möglich. Du musst dich nirgends registrieren.

Wir wünschen dir viel Spaß bei der Umfrage!

Diese Umfrage ist eine Kooperation zwischen der Akademie für Öffentliches Gesundheitswesen (AfÖG), dem Bundesverband der Ärztinnen und Ärzte des öffentlichen Gesundheitsdienstes (BVÖGD), der Bundesvertretung der Medizinstudierenden in Deutschland e.V. (BVMD) und dem Nachwuchsnetzwerk Öffentliche Gesundheit (NÖG).

Anonymität und Datenschutz

Alle Angaben werden selbstverständlich anonym ausgewertet! Um dennoch Aussagen über einzelne Gruppen treffen zu können, benötigen wir ein paar Angaben zu deiner Person. Aus den Ergebnissen werden anschließend **keine Rückschlüsse** auf Einzelpersonen mehr möglich sein. Es geht uns lediglich darum herauszuarbeiten, welche Unterschiede in den beruflichen Erwartungen bestehen.

Wie alt bist du?

Alter in Jahren

[ ]

Biologisches Geschlecht

- ○ männlich
- ○ weiblich
- ○ divers

In welchem Studiengang bist du aktuell primär immatrikuliert (bzw. im Fall von mehreren Studiengängen: Was würdest du als dein Hauptstudium beschreiben)?

- ○ Humanmedizin / Zahnmedizin
- ○ Veterinärmedizin
- ○ Politikwissenschaften
- ○ Psychologie
- ○ Public Health / Gesundheitswissenschaften / Gesundheitsmanagement
- ○ Soziale Arbeit
- ○ Soziologie
- ○ Wirtschaftswissenschaften
- ○ Sonstiges, und zwar: [ ]
- ○ Ich bin derzeit in keinem Studiengang immatrikuliert

In welchem Abschnitt deines Studiums befindest du dich?

- ○ Vorklinischer Teil
- ○ Klinischer Teil
- ○ Modellstudiengang vor dem Praktischen Jahr
- ○ Praktisches Jahr
- ○ Bereits abgeschlossen

Welchen Studienabschluss wirst du aller Voraussicht nach als nächstes erreichen?

- ○ Promotion
- ○ Master
- ○ Bachelor
- ○ Staatsexamen
- ○ Sonstiges, und zwar: ______

Welche Facharztweiterbildungen kommen für dich in Frage?
Mehrfachantworten sind möglich

- ☐ Ich bin aktuell unentschieden
- ☐ Ich strebe aktuell keine Facharztweiterbildung an
- ☐ Innere Medizin
- ☐ Kinder- & Jugendmedizin
- ☐ Allgemeinmedizin
- ☐ Anästhesiologie
- ☐ Chirurgie / Unfallchirurgie / Orthopädie
- ☐ Frauenheilkunde und Geburtshilfe
- ☐ Psychiatrie / Psychotherapie
- ☐ Neurologie
- ☐ Öffentliches Gesundheitswesen
- ☐ Sonstige, und zwar: ______

Hast du vor deinem aktuellen Studium weitere Studiengänge abgeschlossen bzw. bist du aktuell noch in einem weiteren Studiengang immatrikuliert?

- ○ Ja
- ○ Nein

Was für ein Studium hast du bereits abgeschlossen, bzw. in welchem Studiengang bist du gerade zusätzlich eingeschrieben?

- ○ Humanmedizin / Zahnmedizin
- ○ Veterinärmedizin
- ○ Politikwissenschaften
- ○ Psychologie
- ○ Public Health / Gesundheitswissenschaften / Gesundheitsmanagement
- ○ Soziale Arbeit
- ○ Soziologie
- ○ Wirtschaftswissenschaften
- ○ Sonstiges, und zwar __________

In welchem Bereich möchtest du nach dem Studium arbeiten?

Bitte sortiere die Tätigkeiten nach deiner persönlichen Relevanz, wobei „1“ der höchsten Relevanz und „6“ der niedrigsten Relevanz entspricht. Ziehe die einzelnen Tätigkeiten hierzu einfach per Drag&Drop auf den jeweiligen Platz.

- **Klinische bzw. klientenbezogene Tätigkeit (z.B. als praktizierende_r Ärzt_in)**
- **Public Health außerhalb des Öffentlichen Gesundheitsdienstes**
- **Public Health innerhalb des Öffentlichen Gesundheitsdienstes**
- **Forschung und Wissenschaft**
- **Privatwirtschaft**
- **Sonstiges**

1
2
3
4
5
6

An welcher Fakultät absolvierst du dein Medizinstudium?

[Bitte auswählen]

In welchem Bundesland studierst du?

[Bitte auswählen]

Was ist dir in deinem späteren Arbeitsleben besonders wichtig?

	unwichtig	eher unwichtig	weder noch	wichtig	sehr wichtig	keine Angabe
Gute Work-Life-Balance	○	○	○	○	○	○
Vereinbarkeit von Beruf und Familie	○	○	○	○	○	○
Flexible Arbeitszeitgestaltung	○	○	○	○	○	○
Geregelte, regelmäßige Arbeitszeiten (keine Nacht- und Wochenenddienste)	○	○	○	○	○	○
Hohes Gehalt	○	○	○	○	○	○
Arbeitsplatzsicherheit (z.B. unbefristetes Arbeitsverhältnis)	○	○	○	○	○	○
Flache Hierarchiestrukturen	○	○	○	○	○	○
Die Tätigkeit erfordert, sich regelmäßig auf neue Inhalte einlassen zu müssen	○	○	○	○	○	○
Möglichkeit, eigene Ideen einzubringen und eigene Projekte verwirklichen zu können	○	○	○	○	○	○
Die Tätigkeit soll einen möglichst großen gesellschaftlichen Nutzen haben (z.B. möglichst vielen Menschen helfen)	○	○	○	○	○	○
Das Gefühl haben, durch meine Tätigkeit direkt Menschen helfen zu können	○	○	○	○	○	○
Das Gefühl haben, durch meine Tätigkeit die Rahmenbedingungen, in denen Menschen leben und gesundheitliche Entscheidungen treffen, direkt beeinflussen zu können	○	○	○	○	○	○
Möglichkeit, Verantwortung übernehmen zu können (z.B. Personal- oder Projektverantwortung)	○	○	○	○	○	○
Gute berufliche Weiterentwicklungsmöglichkeiten und Aufstiegschancen (z.B. klare Karrierewege)	○	○	○	○	○	○
Möglichkeit, forschend bzw. wissenschaftlich tätig zu sein	○	○	○	○	○	○
Möglichkeit, die eigene Tätigkeit mit einer Promotion zu verbinden	○	○	○	○	○	○
Arbeiten im Team	○	○	○	○	○	○
Arbeiten in einem inter- und multidisziplinären Team	○	○	○	○	○	○
Direkter Kontakt mit Patient_innen / Klient_innen	○	○	○	○	○	○

Möglichkeit, im Ausland tätig zu werden	○	○	○	○	○	○

Dieser Teil des Surveys bezieht sich auf Ansätze die Gesundheit der Bevölkerung zu verbessern, wie diese wahrgenommen werden und ob diese als Arbeitsfeld attraktiv erscheinen oder nicht.

Im Folgenden möchten wir wissen, wie du den aktuellen Stand der Gesundheit sowie deren Entwicklung einschätzt.

Die Auflösung zu diesen Fragen geben wir am Ende des Fragebogens.

	- ≥9 Jahre	- ≥6-8 Jahre	- ≥3-5 Jahre	0 +/- 2 Jahre	+ ≥3-5 Jahre	+ ≥6-8 Jahre	+ ≥9 Jahre
Wie hat sich die durchschnittliche Lebenserwartung in reichen Ländern (d.h. Länder mit einem hohen pro-Kopf Einkommen; z.B. Deutschland) in den vergangenen 30 Jahren verändert?	○	○	○	○	○	○	○
Wie hat sich die durchschnittliche Lebenserwartung in armen Ländern (d.h. Länder mit einem niedrigen pro-Kopf Einkommen; z.B. Indien) in den vergangenen 30 Jahren verändert?	○	○	○	○	○	○	○

	- ≥9 Jahre	- ≥6-8 Jahre	- ≥3-5 Jahre	0 +/- 2 Jahre	+ ≥3-5 Jahre	+ ≥6-8 Jahre	+ ≥9 Jahre
Wie unterscheidet sich die durchschnittliche Lebenserwartung von Frauen in Deutschland mit niedrigem sozioökonomischen Status (SES) von Frauen mit hohem SES?	○	○	○	○	○	○	○
Wie unterscheidet sich die durchschnittliche Lebenserwartung von Männern in Deutschland mit niedrigem sozioökonomischen Status (SES) von Männern mit hohem SES?	○	○	○	○	○	○	○

	- ≥9 Jahre	- ≥6-8 Jahre	- ≥3-5 Jahre	0 +/- 2 Jahre	+ ≥3-5 Jahre	+ ≥6-8 Jahre	+ ≥9 Jahre
Wie unterscheidet sich in Deutschland die durchschnittliche Lebenserwartung von Männern und Frauen?	○	○	○	○	○	○	○

Die Lebenserwartung in Deutschland ist in den letzten 120 Jahren deutlich gestiegen” Welche Rolle spielen hierfür deiner Meinung nach die nachfolgenden Maßnahmen?

Bitte bewerte die Maßnahmen nach deiner Präferenz, wobei „1“ der höchsten Präferenz entspricht und „6“ der niedrigsten Präferenz.

Medizinische Therapie kranker Menschen oder Krankheitsvorsorge bei nicht-übertragbaren Erkrankungen

Medizinische Primärprävention

Medizinische Sekundärprävention

Nicht-medizinische Maßnahmen der Prävention und Gesundheitsförderung

Veränderung von Lebenswelten

Armutsreduktion, Reduktion von Mangelernährung und weitere Maßnahmen

Medizinische Therapie von Infektionskrankheiten

1 – Höchste Relevanz

2

3

4

5

6 – Niedrigste Relevanz

7

Kommen die nachfolgenden Berufsfelder für dich nach deinem Studium in Frage?

	Nein, auf keinen Fall	Nein, eher nicht	Ja, wahr-scheinlich	Ja, auf jeden Fall	keine Angabe
Ausbruchs- und Krisenmanagement	○	○	○	○	○
(Medizinische) Primärprävention und Sekundärprävention	○	○	○	○	○
Verhaltensprävention und Verhältnisprävention	○	○	○	○	○
Gesundheits- und Infektionsschutz	○	○	○	○	○
Gesundheitshilfen für vulnerable Personengruppen	○	○	○	○	○
Gesundheitsplanung und Politikberatung	○	○	○	○	○

Wie intensiv werden bzw. wurden die nachfolgenden Aspekte während deines Hauptstudiums thematisiert?

	nie	selten	manch-mal	oft	sehr oft	bislang noch nicht
Ausbruchs- und Krisenmanagement	○	○	○	○	○	○
(Medizinische) Primärprävention und Sekundärprävention	○	○	○	○	○	○
Verhaltensprävention und Verhältnisprävention	○	○	○	○	○	○
Gesundheits- und Infektionsschutz	○	○	○	○	○	○
Gesundheitshilfen für vulnerable Personengruppen	○	○	○	○	○	○
Gesundheitsplanung und Politikberatung	○	○	○	○	○	○

Der Öffentliche Gesundheitsdienst (ÖGD) stellt neben der ambulanten und stationären Versorgung die dritte Säule des deutschen Gesundheitswesens dar. Er ist dabei für bevölkerungsmedizinische Fragestellungen verantwortlich.

Welche der eben genannten Tätigkeitsfelder gehören deiner Meinung nach in den Aufgabenbereich des Öffentlichen Gesundheitsdienstes (ÖGD)?

	Tätigkeit ist eine Kern-aufgabe des ÖGD	Tätigkeit gehört in den engeren Aufgaben-bereich des ÖGD	Tätigkeit gehört in den weiteren Aufgaben-bereich des ÖGD	Tätigkeit gehört nicht zu dem Aufgaben-bereich des ÖGD	keine Angabe
Ausbruchs- und Krisenmanagement	○	○	○	○	○
(Medizinische) Primärprävention und Sekundärprävention	○	○	○	○	○
Verhaltensprävention und Verhältnisprävention	○	○	○	○	○
Gesundheits- und Infektionsschutz	○	○	○	○	○
Gesundheitshilfen für vulnerable Personengruppen	○	○	○	○	○
Gesundheitsplanung und Politikberatung	○	○	○	○	○

Inwiefern stimmst du den nachfolgenden Aussagen zu?

	Ja	eher Ja	eher Nein	Nein	weiß nicht
Ich weiß, dass es eine Facharztweiterbildung für Öffentliches Gesundheitswesen gibt	○	○	○	○	○
Ich kann mir vorstellen, im öffentlichen Gesundheitsdienst zu arbeiten	○	○	○	○	○
Ich kann mir vorstellen, in einem kommunalen Gesundheitsamt zu arbeiten	○	○	○	○	○
Ich kann mir vorstellen, in einer Gesundheitsbehörde zu arbeiten (z.B. Ministerien, Landesgesundheitsämter, BZgA, RKI)	○	○	○	○	○

Kannst du dir vorstellen einen Studienabschnitt im öffentlichen Gesundheitsdienst zu absolvieren?

Mehrfachantworten sind möglich

- ☐ Ja, im Rahmen eines Wahlfaches
- ☐ Ja, im Rahmen einer Famulatur
- ☐ Ja, im Rahmen eines PJ-Tertials
- ☐ Nein

- ☐ weiß nicht

Nenne drei Adjektive, die du mit dem ÖGD assoziierst

- ☐ ____________________
- ☐ ____________________
- ☐ ____________________

- ☐ Ich kenne den ÖGD nicht

Welche der nachfolgenden Charakteristika assoziierst du mit dem ÖGD?

Die Tätigkeiten im ÖGD sind …

langweilig und eintönig	○	○	○	○	○	interessant und abwechslungsreich
wenig fordernd und fachlich anspruchslos	○	○	○	○	○	herausfordernd und fachlich anspruchsvoll
mit weitreichenden Auswirkungen auf die Gesundheit der Bevölkerung verbunden	○	○	○	○	○	mit geringen Auswirkungen auf die Gesundheit der Bevölkerung verbunden
mit globalen Gesundheitsproblemen verbunden	○	○	○	○	○	mit lokalen Gesundheitsproblemen verbunden
modern und innovativ	○	○	○	○	○	traditionell und veraltet
bürokratisch	○	○	○	○	○	unbürokratisch
mit wenigen Aufstiegschancen verbunden	○	○	○	○	○	mit vielen Aufstiegschancen verbunden
für Personen mit Zielen und Ambitionen	○	○	○	○	○	für Personen, die einen entspannten Job suchen

Der ÖGD wäre für mich ein attraktiver Arbeitgeber...

○ Ja, weil ____________________

○ Nein, weil ____________________

Was müsste geschehen, damit der ÖGD für dich ein attraktiver Arbeitgeber wird?

Möchtest du uns zum Schluss noch etwas mitteilen?

Vielen Dank für deine Teilnahme!

Wir werden versuchen aus den Ergebnissen abzuleiten, welche Weichenstellungen vorgenommen werden müssen um attraktive Jobs und Arbeitsbedingungen zu schaffen. Durch eine Vielzahl an Antworten erhoffen wir uns (politische) Einflussmöglichkeiten, diese Wünsche und Erwartungen auch in deinem und unserem Sinne bei den Arbeitgebern durchzusetzen.

Wenn du die Ergebnisse nutzen möchtest, um dir an deiner Uni oder deinem (zukünftigen) Arbeitsplatz mehr Gehör zu verschaffen, oder wenn du auch nur allgemein daran interessiert bist, halten wir dich gerne über die Ergebnisse der Umfrage auf dem Laufenden.

Als Dankeschön für dein Engagement verlosen wir unter allen Teilnehmenden ein Kongressticket inkl. Übernachtungskosten zum nächsten BVÖGD-Kongress in Saarbrücken (23. – 25. April 2020), ein Lenovo-Tablet und drei Power-Banks.

☐ Ich will am **Gewinnspiel** teilnehmen. Ich willige ein, dass meine E-Mail-Adresse bis zur Ziehung der Gewinner gespeichert wird. Diese Einwilligung kann ich jederzeit widerrufen. Meine Angaben in dieser Befragung bleiben weiterhin anonym, meine E-Mail-Adresse wird nicht an Dritte weitergegeben.

☐ Ich interessiere mich für die **Ergebnisse dieser Studie** und hätte gerne eine Zusammenfassung per E-Mail.

Vielen Dank für deine Unterstützung!

Diese Umfrage ist eine Kooperation zwischen der Akademie für Öffentliches Gesundheitswesen (AfÖG), dem Bundesverband der Ärztinnen und Ärzte des öffentlichen Gesundheitsdienstes (BVÖGD), der Bundesvertretung der Medizinstudierenden in Deutschland e.V. (bvmd) und dem Nachwuchsnetzwerk Öffentliche Gesundheit (NÖG)

Akademie für Öffentliches Gesundheitswesen in Düsseldorf · bvmd Bundesvertretung der Medizinstudierenden in Deutschland e.V. German Medical Students' Association · BVÖGD Bundesverband der Ärztinnen und Ärzte des Öffentlichen Gesundheitsdienstes e.V. · NÖG Nachwuchsnetzwerk Öffentliche Gesundheit

Hier geht es zur Auflösung der Public Health-Fragen.

Deine Antworten wurden gespeichert, du kannst das Browser-Fenster nun schließen.

Möchten Sie in Zukunft an interessanten und spannenden Online-Befragungen teilnehmen?

Wir würden uns sehr freuen, wenn Sie Ihre E-Mail-Adresse für das SoSci Panel anmelden und damit wissenschaftliche Forschungsprojekte unterstützen.

E-Mail: [ ] Am Panel teilnehmen

Die Teilnahme am SoSci Panel ist freiwillig, unverbindlich und kann jederzeit widerrufen werden.
Das SoSci Panel speichert Ihre E-Mail-Adresse nicht ohne Ihr Einverständnis, sendet Ihnen keine Werbung und gibt Ihre E-Mail-Adresse nicht an Dritte weiter.

Sie können das Browserfenster selbstverständlich auch schließen, ohne am SoSci Panel teilzunehmen.
