# Supplementary material for: What Factors Influence the Interest in Working in the Public Health Service in Germany? Part I of the OeGD-Studisurvey
Source: Int J Environ Res Public Health. 2022 Sep 19;19(18):11838. doi: 10.3390/ijerph191811838 (PMC9517554; doi:10.3390/ijerph191811838)
Supplement: Supplementary file 1 [file ijerph-19-11838-s001.zip › S2_OeGD-Studisurvey_PartI_Questionaire_wave2.pdf]

Herzlich willkommen!

Wir freuen uns, dass du dir kurz Zeit für unsere Umfrage nimmst!

Mit diesem Survey wollen wir herausfinden, (1) welche **Wünsche und Ansprüche** Studierende und Nachwuchskräfte im Bereich Public Health/Öffentliche Gesundheit an ihre zukünftige Arbeit und ihre Arbeitgeber:innen haben und (2) was das konkret für die Weiterentwicklung von **Ausbildungs- und Karrierewege** bedeutet. In dieser Umfrage steht der öffentliche Gesundheitsdienst (ÖGD) als ein mögliches Berufsfeld im Vordergrund.

Wir haben diese Umfrage in sehr ähnlicher Form bereits Anfang 2020 durchgeführt. Da sich durch die Corona-Pandemie sehr viel verändert hat, möchten wir euch gerne erneut um eure Einschätzung bitten. Der ÖGD erfährt derzeit so viel mediale und politische Aufmerksamkeit, dass es ein sehr guter Zeitpunkt für Veränderungen ist.

Je mehr Personen teilnehmen, desto eher können wir aus den Ergebnissen ableiten, welche **Weichenstellungen** vorgenommen werden müssen, um attraktive Jobs und Arbeitsbedingungen im Public Health-Sektor allgemein und speziell im ÖGD zu schaffen. Zum anderen verleiht uns (und dir) eine große Zahl von Antworten mehr **(politische) Einflussmöglichkeiten**, diese Wünsche und Erwartungen auch bei zukünftigen Arbeitgeber:innen durchzusetzen.

Dabei gilt selbstverständlich: Unsere Befragung ist **anonym**! Wir fragen ein paar wenige personenbezogene Daten ab (z.B. was du studierst), aber du als Individuum bist nicht identifizierbar und Rückschlüsse auf die jeweilige Person sind zu keinem Zeitpunkt möglich. Du musst dich auch nirgends registrieren.

Wir danken dir für dein Engagement und wünschen dir viel Spaß bei der Umfrage!

Dieser Survey wird vom Nachwuchsnetzwerk Öffentliche Gesundheit (NÖG) durchgeführt. Die Befragung wurde in etwas anderer Form bereits im Jahr 2019/2020 als Kooperation zwischen der Akademie für Öffentliches Gesundheitswesen (AÖGW), dem Bundesverband der Ärztinnen und Ärzte des Öffentlichen Gesundheitsdienstes e.V. (BVÖGD), der Bundesvertretung der Medizinstudierenden in Deutschland e.V. (BVMD) und dem Nachwuchsnetzwerk Öffentliche Gesundheit (NÖG) durchgeführt.

Anonymität und Datenschutz

Alle Angaben werden selbstverständlich anonym ausgewertet! Um dennoch Aussagen über einzelne Personengruppen treffen zu können, benötigen wir ein paar wenige Angaben zu deiner Person. Aus den Ergebnissen werden anschließend **keine Rückschlüsse** auf dich mehr möglich sein. Es geht uns lediglich darum herauszuarbeiten, welche Unterschiede in den beruflichen Erwartungen bestehen.

Die Angaben zu deiner Person werden ausschließlich im Rahmen dieser Studie verwendet. Dazu gehören personenbezogene Daten wie z.B. Alter, Geschlecht und Bildungsabschluss. Die Daten werden anonymisiert erhoben – dies schließt eine Identifikation einzelner Personen auf Basis der Daten weitgehend aus. Zu der anonymen Datenerhebung kannst du beitragen, indem du beim Ausfüllen der Freitextfelder darauf achtest, keine Informationen über dich mitzuteilen, die einen direkten Rückschluss auf dich zulassen (bspw. keine Nennung aktueller Arbeitgeber:innen). Deine Daten werden verschlüsselt und passwortgeschützt gespeichert und nicht an Dritte weitergegeben. Die Daten werden gemäß der DFG-Empfehlungen zehn Jahre nach ihrer Erhebung gelöscht.

Die Befragung wird über die Onlineplattform SoSci Survey durchgeführt, welche sich durch ein hohes Datenschutzniveau auszeichnet (Link). Die Einwilligung zur Verarbeitung deiner Daten ist freiwillig.

Für Rückfragen zu der Umfrage inklusive des Datenschutzes stehen wir jederzeit unter umfrage@noeg.org zur Verfügung.

Einwilligung Datenschutz

- ☐ Ich erkläre, dass ich bereit bin, an der Umfrage teilzunehmen. Ich bin mit der Verarbeitung meiner Daten im Rahmen dieser Umfrage einverstanden
- ☐ Ich möchte an der Umfrage nicht teilnehmen

Wie alt bist du?

Alter in Jahren

[ ]

Geschlecht

- ○ männlich
- ○ weiblich
- ○ divers
- ○ keine Angabe

In welchem Studiengang bist du aktuell primär immatrikuliert?

Bzw. im Fall von mehreren Studiengängen: Was würdest du als dein Hauptstudium beschreiben?

- ○ Humanmedizin / Zahnmedizin
- ○ Veterinärmedizin
- ○ Public Health / Gesundheitswissenschaften / Gesundheitsmanagement (oder ein vergleichbares Fach)
- ○ Sozial- / Politik- Wirtschafts- / Verwaltungswissenschaften (oder ein vergleichbares Fach)
- ○ Psychologie
- ○ Sonstiges, und zwar: [ ]
- ○ Ich bin derzeit in keinem Studiengang immatrikuliert

In welchem Abschnitt deines Studiums befindest du dich?

- ○ Vorklinischer Teil
- ○ Klinischer Teil
- ○ Modellstudiengang vor dem Praktischen Jahr
- ○ Praktisches Jahr
- ○ Bereits abgeschlossen

Welchen Studienabschluss wirst du aller Voraussicht nach als nächstes erreichen?

- ○ Bachelor
- ○ Master
- ○ Staatsexamen
- ○ Promotion
- ○ Sonstiges, und zwar: ________

Welche Facharztweiterbildungen kommen für dich in Frage?

Mehrfachantworten sind möglich

- ☐ Ich bin aktuell unentschieden
- ☐ Ich strebe aktuell keine Facharztweiterbildung an
- ☐ Innere Medizin
- ☐ Kinder- & Jugendmedizin
- ☐ Allgemeinmedizin
- ☐ Anästhesiologie
- ☐ Chirurgie / Unfallchirurgie / Orthopädie
- ☐ Frauenheilkunde und Geburtshilfe
- ☐ Psychiatrie / Psychotherapie
- ☐ Neurologie
- ☐ Öffentliches Gesundheitswesen
- ☐ Sonstige, und zwar: ________

Hast du vor deinem aktuellen Studium weitere Studiengänge abgeschlossen bzw. bist du aktuell noch in einem weiteren Studiengang immatrikuliert?

- ○ Ja
- ○ Nein

Was für ein Studium hast du bereits abgeschlossen, bzw. in welchem Studiengang bist du gerade zusätzlich eingeschrieben?

- ○ Humanmedizin / Zahnmedizin
- ○ Veterinärmedizin
- ○ Public Health / Gesundheitswissenschaften / Gesundheitsmanagement (oder vergleichbares Fach)
- ○ Sozial- / Politik- / Wirtschafts-/ Verwaltungswissenschaften (oder vergleichbares Fach)
- ○ Psychologie
- ○ Sonstiges, und zwar ____________

Kommt eine berufliche Tätigkeit in einem der folgenden Bereiche für dich in Frage?

	Nein, auf keinen Fall	Nein, eher nicht	Ja, wahr-scheinlich	Ja, auf jeden Fall	Keine Angabe
Klinische Tätigkeit (z.B. als praktizierende:r Ärzt:in)	○	○	○	○	○
Public Health innerhalb des Öffentlichen Gesundheitsdienstes auf kommunaler Ebene (z.B. Gesundheitsämter)	○	○	○	○	○
Public Health innerhalb des Öffentlichen Gesundheitsdienstes auf Landesebene (z.B. Landesgesundheitsämter)	○	○	○	○	○
Public Health innerhalb des Öffentlichen Gesundheitsdienstes auf Bundesebene (z.B. Robert Koch-Institut, Paul Ehrlich-Institut, Bundeszentrale für gesundheitliche Aufklärung)	○	○	○	○	○
Public Health außerhalb des Öffentlichen Gesundheitsdienstes (z.B. Sozialversicherungsträger, NGOs, Betriebliches Gesundheitsmanagement, Vereine)	○	○	○	○	○
Public Health in internationalen Institutionen (z.B. Weltgesundheitsorganisation, EU Gesundheitsbehörde)	○	○	○	○	○
Forschung und Wissenschaft (innerhalb oder außerhalb von Universitäten und Hochschulen)	○	○	○	○	○
Privatwirtschaft (z.B. Consulting, Produktentwicklung; ohne Betriebliches Gesundheitsmanagement)	○	○	○	○	○

An welcher Fakultät absolvierst du dein Medizinstudium?

[Bitte auswählen]

In welchem Bundesland studierst du?

[Bitte auswählen]

Was ist dir in deinem späteren Arbeitsleben besonders wichtig?

Bitte sortiere die Tätigkeiten nach deiner persönlichen Relevanz, wobei „1“ der höchsten Relevanz und „11“ der niedrigsten Relevanz entspricht. Klicke die einzelnen Charakeristika hierzu einfach in aufsteigender Reihenfolge an. Beginne mit der höchsten Relevanz. Falls du Einträge nachträglich noch ändern möchtest, kannst du das jederzeit durch einen zweiten Klick auf das jeweilige Ranking tun.

- Work-Life-Balance (u.a. Vereinbarkeit Beruf und Familie)
- Flexible Arbeitszeitgestaltung
- Hohes Gehalt
- Arbeitsplatzsicherheit (z.B. Entfristung, Verbeamtung)
- Gesellschaftliche Anerkennung und Reputation
- Wertschätzendes und angenehmes Arbeitsklima
- Abwechslungsreiche Tätigkeit mit Gestaltungsspielraum
- Tätigkeit mit großem gesellschaftlichem Nutzen
- Direkter Kontakt mit Klient:innen und Patient:innen
- Aufstiegschancen und Möglichkeit zur Weiterbildung
- Forschende bzw. wissenschaftliche Tätigkeit

1
2
3
4
5
6
7
8
9
10
11

Kommen die nachfolgenden Tätigkeiten und Aufgabengebiete für dich nach deinem Studium in Frage?

	Nein, auf keinen Fall	Nein, eher nicht	Ja, wahr-scheinlich	Ja, auf jeden Fall	keine Angabe
Erstellung personenbezogener Gutachten und Stellungnahmen	○	○	○	○	○
Beratung zu Themen der Gesundheitsförderung, Krankheitsprävention, Rechten und Unterstützungsangeboten	○	○	○	○	○
Durchführung von ärztlichen Anamnesegesprächen und medizinischen Untersuchungen	○	○	○	○	○
Untersuchung potentieller Ausbrüche von Infektionskrankheiten, Untersuchung des Ausbruchsgeschehens inkl. Veranlassung und Durchführung notwendiger Maßnahmen	○	○	○	○	○
Beratung zum Thema Impfen oder Reisemedizin inkl. Durchführung von Impfungen	○	○	○	○	○
Planung, Organisation und Durchführung von Projekten der Gesundheitsförderung oder Krankheitsprävention	○	○	○	○	○
Gesundheits- und Risikokommunikation, z.B. in Form von Planung und Durchführung von Maßnahmen mit dem Ziel, über gesundheitsrelevante Sachverhalte aufzuklären	○	○	○	○	○
Auswertung vorhandene bzw. Generierung neuer Daten, Analyse und Aufbereitung selbiger mit dem Ziel, Entscheidungsträger*Innen informiertes Handeln zu ermöglichen	○	○	○	○	○
Beratung von politischen Entscheidungsträger:innen zu gesundheitsrelevanten Sachverhalten	○	○	○	○	○
Durchführung von Routineuntersuchungen in einzelnen Settings	○	○	○	○	○
Analytisch-technische Labortätigkeit inkl. Auswertung und Interpretation von Befunden	○	○	○	○	○
Arbeit mit vulnerablen Personengruppen	○	○	○	○	○
Beratung bei psychischer Erkrankung	○	○	○	○	○
Beratung, Überwachung und aktive Untersuchung von Elementen der gebauten und natürlichen Umwelt	○	○	○	○	○
Beratung bei Hygienefragen oder bei der Überwachung von medizinischen Einrichtungen	○	○	○	○	○

Ich habe bereits Berufserfahrungen im ÖGD gesammelt

Mehrfachnennungen sind möglich

- ☐ Ja, im Rahmen meines Studiums
- ☐ Ja, im Rahmen meiner beruflichen Tätigkeit
- ☐ Ja, im Rahmen von ______
- ☐ Nein

- ☐ weiß nicht

Ich kann mir vorstellen, eine Facharztweiterbildung für das Öffentliche Gesundheitswesen zu machen

- ○ Nein, auf keinen Fall
- ○ Nein, eher nicht
- ○ Ja, wahrscheinlich
- ○ Ja, auf jeden Fall

- ○ weiß nicht

Wenn es eine Art “Facharztweiterbildung” für das Öffentliche Gesundheitswesen mit entsprechenden Berufs- und Karriereperspektiven auch für qualifizierte Personen ohne Medizinstudium geöffnet wäre (z.B. ähnlich zum britischen Modell), könnte ich mir vorstellen eine solche Ausbildung zu machen

- ○ Nein, auf keinen Fall
- ○ Nein, eher nicht
- ○ Ja, wahrscheinlich
- ○ Ja, auf jeden Fall

- ○ weiß nicht

Ich weiß, dass es eine Facharztweiterbildung für Öffentliches Gesundheitswesen gibt

- ○ Ja
- ○ Nein

Ich kann mir vorstellen, im Öffentlichen Gesundheitsdienst tätig zu sein

- ○ Ja, auf jeden Fall
- ○ Ja, wahrscheinlich
- ○ Nein, eher nicht
- ○ Nein, auf keinen Fall

- ○ weiß nicht

Welche der folgenden Tätigkeiten im ÖGD kannst du dir vorstellen?

	Nein, auf keinen Fall	Nein, eher nicht	Ja, wahr-schein-lich	Ja, auf jeden Fall	weiß nicht
Ich kann mir vorstellen, in einem kommunalen Gesundheitsamt zu arbeiten	○	○	○	○	○
Ich kann mir vorstellen, in einer mittleren Gesundheitsbehörde zu arbeiten	○	○	○	○	○
Ich kann mir vorstellen, in einer oberen Gesundheitsbehörde zu arbeiten	○	○	○	○	○

Kannst du dir vorstellen einen Studienabschnitt im öffentlichen Gesundheitsdienst zu absolvieren?

- ○ Ja, im Rahmen eines Wahlfaches
- ○ Ja, im Rahmen einer Famulatur
- ○ Ja, im Rahmen eines PJ-Tertials
- ○ Sonstiges, und zwar: __________
- ○ Nein

- ○ weiß nicht

Kannst du dir vorstellen einen Studienabschnitt im öffentlichen Gesundheitsdienst zu absolvieren?

- ○ Ja, im Rahmen eines Pflichtpraktikums
- ○ Ja, im Rahmen einer freiwilligen Praktikums
- ○ Ja, im Rahmen eines studienbegleitenden Praktikums
- ○ Ja, im Rahmen einer Projektarbeit
- ○ Ja, im Rahmen einer Abschlussarbeit
- ○ Sonstiges, und zwar:
- ○ Nein

- ○ weiß nicht

Nenne drei Charakteristika, die du mit dem ÖGD assoziierst

- ☐ ______
- ☐ ______
- ☐ ______

Welche der nachfolgenden Charakteristika assoziierst du mit dem ÖGD?

Die Tätigkeiten im ÖGD sind …

interessant und abwechslungsreich	○	○	○	○	○	langweilig und eintönig
herausfordernd und fachlich anspruchsvoll	○	○	○	○	○	wenig fordernd und fachlich anspruchslos
mit weitreichenden Auswirkungen auf die Gesundheit der Bevölkerung verbunden	○	○	○	○	○	mit geringen Auswirkungen auf die Gesundheit der Bevölkerung verbunden
mit lokalen Gesundheitsproblemen verbunden	○	○	○	○	○	mit globalen Gesundheitsproblemen verbunden
modern und innovativ	○	○	○	○	○	traditionell und veraltet
bürokratisch	○	○	○	○	○	unbürokratisch
mit wenigen Aufstiegschancen verbunden	○	○	○	○	○	mit vielen Aufstiegschancen verbunden
für Personen, die einen entspannten Job suchen	○	○	○	○	○	für Personen mit Zielen und Ambitionen

Welche der nachfolgenden Charakteristika assoziierst du mit einer ÖGD-Tätigkeit auf kommunaler Ebene (z.B. Arbeit im Gesundheitsamt)?

Die Tätigkeiten im kommunalen ÖGD sind …

langweilig und eintönig	○	○	○	○	○	interessant und abwechslungsreich
herausfordernd und fachlich anspruchsvoll	○	○	○	○	○	wenig fordernd und fachlich anspruchslos
irrelevant für die Bevölkerungsgesundheit	○	○	○	○	○	relevant für die Bevölkerungsgesundheit
traditionell und veraltet	○	○	○	○	○	modern und innovativ
verwaltend	○	○	○	○	○	gestaltend
mit wenigen Aufstiegschancen verbunden	○	○	○	○	○	mit vielen Aufstiegschancen verbunden
für Personen mit Zielen und Ambitionen	○	○	○	○	○	für Personen, die einen entspannten Job suchen
mit einer schlechten Work-Life Balance verbunden	○	○	○	○	○	mit einer guten Work-Life Balance verbunden

Welche der nachfolgenden Charakteristika assoziierst du mit einer ÖGD-Tätigkeit auf Landes- oder Bundesebene (z.B. Arbeit in einem Landesgesundheitsamt oder einer Bundesbehörde)?

Public Health auf Landes- oder Bundesebene ist …

langweilig und eintönig	○	○	○	○	○	interessant und abwechslungsreich
wenig fordernd und fachlich anspruchslos	○	○	○	○	○	herausfordernd und fachlich anspruchsvoll
relevant für die Bevölkerungsgesundheit	○	○	○	○	○	irrelevant für die Bevölkerungsgesundheit
traditionell und veraltet	○	○	○	○	○	modern und innovativ
gestaltend	○	○	○	○	○	verwaltend
mit vielen Aufstiegschancen verbunden	○	○	○	○	○	mit wenigen Aufstiegschancen verbunden
für Personen mit Zielen und Ambitionen	○	○	○	○	○	für Personen, die einen entspannten Job suchen
mit einer guten Work-Life Balance verbunden	○	○	○	○	○	mit einer schlechten Work-Life Balance verbunden

Der ÖGD wäre für mich ein attraktiver Arbeitgeber...

- ○ Ja, weil ____________
- ○ Nein, weil ____________

Was müsste geschehen, damit der ÖGD für dich ein attraktiver Arbeitgeber wird?

Mit welchen Themen müsste sich der ÖGD auf <u>kommunaler Ebene</u> beschäftigen, um ein attraktiverer Arbeitgeber zu sein? Welche Tätigkeiten und Aufgaben gehen für dich damit einher?

Mit welchen Themen müsste sich der ÖGD auf <u>Landes- und Bundesebene</u> beschäftigen, um ein attraktiverer Arbeitgeber zu sein? Welche Tätigkeiten und Aufgaben gehen für dich damit einher?

Welche Aufgabenbereiche sollte der ÖGD deiner Meinung im Vergleich zur derzeitigen Situation in Zukunft mehr oder weniger angehen?

Verschiebe hierzu bitte den Schieberegeler für jeden einzelnen Aufgabenbereich entsprechend deiner Einschätzung.

	weniger	mehr
Sicherstellung des Gesundheits- und Infektionsschutzes		
Gesundheitshilfen für vulnerable Personengruppen		
Reaktion auf ökonomische wie ökologische Herausforderungen (z.B. im Kontext des Klimawandels)		
Mitarbeit bei der Stadt- und Kommunalentwicklung		
Initiierung von Programmen zur Krankheitsprävention und Gesundheitsförderung		
Multiplikatorenrolle: Netzwerk- und Moderatorenfunktion		
Übernahme von Koordinations- und Steuerungsaufgaben		
Politikberatung		
Information der Bevölkerung und (Fach-)Öffentlichkeit		
Datenanalyse und Berichterstattung		
Sozialen wie gesundheitlichen Ungleichheiten entgegenwirken		
Gutachtertätigkeiten		
Beratertätigkeiten		
Kontrollen zur Sicherstellung von Qualitätsstandards		

Der ÖGD sollte darüber hinaus zukünftig folgende Aufgabenbereiche abdecken:

Im Folgendenden geht es um eine Tätigkeit im ÖGD während der Corona-Pandemie

	Nein, auf keinen Fall	Nein, eher nicht	Ja, wahr-schein-lich	Ja, auf jeden Fall	weiß nicht
Hättest du dir **vor** der Pandemie vorstellen können im ÖGD zu arbeiten?	○	○	○	○	○
Hättest du dir **während** der Pandemie vorstellen können im ÖGD zu arbeiten?	○	○	○	○	○
Könntest du dir **nach** der Pandemie vorstellen im ÖGD zu arbeiten?	○	○	○	○	○

Falls sich deine Einschätzung über die Zeit verändert hat, warum war das der Fall?

Hast du vom ÖGD-Pakt gehört?

- ○ Ja
- ○ Nein

Zur nachhaltigen Stärkung des ÖGD haben Bund und Länder am 29. September 2020 den insgesamt 4 Mrd. EURO schweren Pakt für den ÖGD beschlossen. Im Mittelpunkt stehen dabei die Themen Personalaufbau, die Steigerung der Attraktivität des ÖGD, den Ausbau der Digitalisierung sowie die Förderung zukunftsfähiger Strukturen innerhalb des ÖGD. Ein besonderes Augenmerk liegt dabei auf den kommunalen Gesundheitsämtern.

Der Pakt bietet aber nicht nur die Gelegenheit, die Gesundheitsämter in Deutschland personell wie informationstechnisch für die Bewältigung der aktuellen oder zukünftiger Pandemien zu stärken, sondern könnte bestenfalls auch die Grundlage für eine nachhaltige Modernisierung legen.

Was erhoffst du dir von dem ÖGD-Pakt für die Weiterentwicklung des ÖGD? Was sollte auf jeden Fall passieren? Was sollte keinesfalls passieren?

Wie sollte der ÖGD der Zukunft deiner Meinung nach aussehen? Was wäre ein best case-Szenario? Was wäre ein worst case-Szenario?

Möchtest du uns zum Schluss noch etwas mitteilen?

Vielen Dank für deine Unterstützung!

Zusammenfassung der Studienergebnisse per E-Mail

☐ Ich interessiere mich für die Ergebnisse dieser Studie und hätte gerne eine Zusammenfassung per E-Mail. Ich willige dazu ein, dass meine E-Mail-Adresse hierfür gespeichert wird. Diese Einwilligung kann ich jederzeit widerrufen. Meine Angaben in dieser Befragung bleiben weiterhin anonym, meine E-Mail-Adresse wird nicht an Dritte weitergegeben.

Deine Kontaktdaten werden ausschließlich für die Weitergabe der Studienergebnisse im obigen Sinne genutzt. Deine E-Mail-Adresse wird hierzu getrennt von den anderen Angaben gespeichert. Es werden keinerlei weitere personenbezogene Daten erhoben.

Deine Antworten wurden gespeichert, du kannst das Browser-Fenster nun schließen.

Möchten Sie in Zukunft an interessanten und spannenden Online-Befragungen teilnehmen?

Wir würden uns sehr freuen, wenn Sie Ihre E-Mail-Adresse für das SoSci Panel anmelden und damit wissenschaftliche Forschungsprojekte unterstützen.

E-Mail: ____________________ [Am Panel teilnehmen]

Die Teilnahme am SoSci Panel ist freiwillig, unverbindlich und kann jederzeit widerrufen werden.
Das SoSci Panel speichert Ihre E-Mail-Adresse nicht ohne Ihr Einverständnis, sendet Ihnen keine Werbung und gibt Ihre E-Mail-Adresse nicht an Dritte weiter.

Sie können das Browserfenster selbstverständlich auch schließen, ohne am SoSci Panel teilzunehmen.
